# Magmatic Response to Subduction Initiation: Part 1. Fore‐arc Basalts of the Izu‐Bonin Arc From IODP Expedition 352

**DOI:** 10.1029/2018GC007731

**Published:** 2019-01-16

**Authors:** John W. Shervais, Mark Reagan, Emily Haugen, Renat R. Almeev, Julian A. Pearce, Julie Prytulak, Jeffrey G. Ryan, Scott A. Whattam, Marguerite Godard, Timothy Chapman, Hongyan Li, Walter Kurz, Wendy R. Nelson, Daniel Heaton, Maria Kirchenbaur, Kenji Shimizu, Tetsuya Sakuyama, Yibing Li, Scott K. Vetter

**Affiliations:** ^1^ Department of Geology Utah State University Logan UT USA; ^2^ Department of Earth and Environmental Science University of Iowa Iowa City IA USA; ^3^ Now at Department of Geology California State University Sacramento CA USA; ^4^ Institut für Mineralogie Leibniz Universität Hannover Hannover Germany; ^5^ School of Earth and Ocean Sciences Cardiff University Cardiff UK; ^6^ Department of Earth Sciences University of Durham Durham UK; ^7^ School of Geosciences University of South Florida Tampa FL USA; ^8^ Department of Geosciences King Fahd University of Petroleum and Minerals Dhahran Saudi Arabia; ^9^ Géosciences Montpellier, CNRS Université de Montpellier Montpellier France; ^10^ School of Geosciences University of Sydney Sydney New South Wales Australia; ^11^ State Key Laboratory of Isotope Geochemistry, Guangzhou Institute of Geochemistry Chinese Academy of Sciences Guangzhou China; ^12^ Institute of Earth Sciences, NAWI Graz Geocenter University of Graz Graz Austria; ^13^ Department of Physics, Astronomy, and Geosciences Towson University Towson MD USA; ^14^ CEOAS Oregon State University Corvallis OR USA; ^15^ Institut für Mineralogie Universität zu Köln Köln Germany; ^16^ Japan Agency for Marine‐Earth Science and Technology Kochi Institute for Core Sample Research Kochi Japan; ^17^ Department of Science Osaka University Osaka Japan; ^18^ Chinese Academy of Geological Science Institute of Geology Beijing China; ^19^ Department of Geology Centenary College Shreveport LA USA

**Keywords:** Forearc basalts, subduction initiation, ophiolites, Izu‐Bonin forearc, Joides Resolution

## Abstract

The Izu‐Bonin‐Mariana (IBM) fore arc preserves igneous rock assemblages that formed during subduction initiation circa 52 Ma. International Ocean Discovery Program (IODP) Expedition 352 cored four sites in the fore arc near the Ogasawara Plateau in order to document the magmatic response to subduction initiation and the physical, petrologic, and chemical stratigraphy of a nascent subduction zone. Two of these sites (U1440 and U1441) are underlain by fore‐arc basalt (FAB). FABs have mid‐ocean ridge basalt (MORB)‐like compositions, however, FAB are consistently lower in the high‐field strength elements (TiO_2_, P_2_O_5_, Zr) and Ni compared to MORB, with Na_2_O at the low end of the MORB field and FeO* at the high end. Almost all FABs are light rare earth element depleted, with low total REE, and have low ratios of highly incompatible to less incompatible elements (Ti/V, Zr/Y, Ce/Yb, and Zr/Sm) relative to MORB. Chemostratigraphic trends in Hole U1440B are consistent with the uppermost lavas forming off axis, whereas the lower lavas formed beneath a spreading center axis. Axial magma of U1440B becomes more fractionated upsection; overlying off‐axis magmas return to more primitive compositions. Melt models require a two‐stage process, with early garnet field melts extracted prior to later spinel field melts, with up to 23% melting to form the most depleted compositions. Mantle equilibration temperatures are higher than normal MORB (1,400 °C–1,480 °C) at relatively low pressures (1–2 GPa), which may reflect an influence of the Manus plume during subduction initiation. Our data support previous models of FAB origin by decompression melting but imply a source more depleted than normal MORB source mantle.

## Key Points

- Fore‐arc basalts (FABs) formed by decompression melting in response to subduction initiation and differ from mid‐ocean ridge basalts
- FABs form by two‐stage melting, with early garnet field melts extracted prior to spinel field melting, resulting in LREE/HREE depletion
- Highly depleted FAB may reflect both an older depletion event and higher ambient temperatures related to the Manus plume

## 1 Introduction

Subduction initiation is a singular and remarkable event in the life of a convergent margin and one that is necessary for plate tectonics to exist (e.g., Wakabayashi & Shervais, 2012). It has long been known that plate motions are driven largely (~90%) by slab pull from subducting oceanic lithosphere (e.g., Forsyth & Uyeda, 1975), but this driving force does not exist until stable subduction is achieved. Initiating subduction in the absence of slab pull is challenging and requires special geodynamic circumstances, for example, nucleation on preexisting zones of weakness, gravitation failure of the slab, and far‐field geodynamic forces (e.g., Crameri et al., 2012 *;* Gurnis et al., 2004; Hall, 2002; Hall et al., 2003 *;* Leng et al., 2012 *;* Stern, 2004; Stern & Gerya, 2017). The most commonly proposed preexisting zones of weakness include fracture zones (Choi et al., 2008; Gurnis et al., 2004; Shervais & Choi, 2012; Stern et al., 2012; Stern & Bloomer, 1992) and oceanic detachments (Maffione et al., 2015; van Hinsbergen et al., 2015). Stern and Bloomer (1992) proposed that subduction may initiate by gravitation failure spontaneously across fracture zones when one side is much older (denser) than the other, but modeling suggests that even in this situation, significant convergence is required before failure occurs (Gurnis et al., 2004; Hall et al., 2003; Leng et al., 2012; Schmeling et al., 2008).

The type locality for many of these models is the Izu‐Bonin‐Mariana (IBM) subduction system in the western Pacific Ocean (Bloomer et al., 1995; Ishizuka et al., 2006; Reagan et al., 2013; Stern & Bloomer*,* 1992). Subduction began here in the early Eocene, possibly coincident with both the Hawaii‐Emperor chain inflection and the collision of India with mainland Asia (e.g., Meade, 2007; Najman et al., 2010; Torsvik et al., 2017; Whittaker et al., 2007). Its intraoceanic location and lack of collisional deformation make it ideal for understanding the magmatic response to subduction initiation and the paragenesis of these magmas. Early work suggested that the initial melts were boninitic, based on the ubiquitous occurrence of boninite series lavas in the IBM fore arc, their highly depleted compositions, and the characteristic presence of clinoenstatite, which implied high fractions of melt extraction from a harzburgitic protolith during water saturated melting (Cameron, 1985; Crawford et al., 1989 *;* Pearce et al., 1992). In addition, the IBM boninites are Eocene in age, older than other volcanic rocks of the IBM system *(*Ishizuka et al., 2006; Meijer et al., 1983).

Reagan et al. (2010) discovered that mid‐ocean ridge basalt (MORB)‐like basalts and related intrusive rocks were the most abundant igneous rocks in the Mariana fore arc. They contended that these basalts and similar lavas found in the Izu (DeBari et al., 1999) and Bonin (Ishizuka, Taylor, et al., 2011) fore arcs were the oldest igneous rocks in the IBM subduction system and were likely generated during fore‐arc spreading in the immediate aftermath of subduction initiation. They proposed a new class of volcanic rocks called *fore‐arc basalts* (FABs) because of their presence in the modern fore arc and to distinguish them from later arc and back‐arc basalts and from mid‐ocean ridge tholeiites, with which they share many similarities. In the IBM fore arc, these basalts occur stratigraphically below the boninite sequences (Reagan et al., 2010), and their Ar‐Ar ages are <2 Ma older than the associated boninites (Cosca et al., 1998; Ishizuka et al., 2006; Ishizuka, Taylor, et al., 2011; Reagan et al., 2013 *)*. This discovery supported the observation of Shervais (2001), who proposed that those ophiolites thought to represent nascent subduction zones exhibit a volcanic stratigraphy marked by early tholeiitic basalt, followed by boninitic magmas, and then by normal arc tholeiite and/or calc‐alkaline suites. Despite the difference in their tectonic settings, the close affinities between FAB and MORB make constraining FAB petrogenesis critical for understanding subduction initiation and the origin of *suprasubduction zone* ophiolites (e.g., Pearce et al., 1984, 1992; Shervais, 2001; Pearce & Robinson, 2010; Stern et al., 2012; Whattam & Stern, 2011).

We present here new data on the petrogenesis of FAB sampled by IODP (International Ocean Discovery Program) Expedition 352 (Reagan et al., 2015a, 2017). Two holes—U1440 and U1441—are composed almost entirely of FAB, with minor FAB‐related basaltic andesite. In this paper, we document the chemostratigraphy of core from these holes, the geochemical evolution of FAB, and present petrogenetic models for its origins.

## 2 Geologic Setting

**Figure 1:**
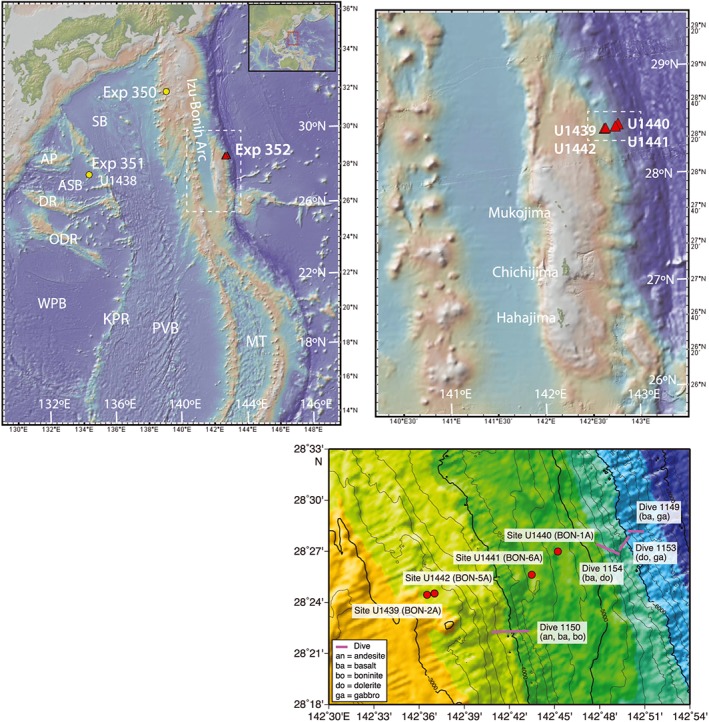
Location maps for IODP Expedition 352. (a) The Izu‐Bonin‐Marian arc system along the western Pacific margin and the Philippine Sea plate back‐arc basins. Red triangles show location of the Expedition 352 drill sites; yellow circles show drill sites for sister expeditions 350 and 351. KPR = Kyushu‐Palau Ridge; PVB = Parece‐Vela Basin; MT = Marian Trough, WPB = West Philippine Basin, SB = Shikoku Basin, AB = Amami Plateau, ASB = Amami‐Sankaku Basin, DR = Daito Ridge, ODR = Oki‐Daito Ridge. Dashed inset shows area of Figure 2b. (b) Detail of the Bonin arc segment, showing arc volcanoes on the left (west), the Izu‐Bonin trench on the right (east), with the Ogasawara Plateau with the islands Mukojima, Chichijima, and Hahajima. Sites U1440 and U1441 are closest to the trench, whereas Sites U1439 and U1442 are farther upslope. Inset shows area of Figure 2c. (c) Detailed topographic map of the inner trench wall showing drill site locations for Sites U1439, U1440, U1441, and U1442. BON‐1A, BON‐2A, BON‐5A, and BON‐6A refer to site designations in the Expedition 352 drilling prospectus. Map colors: purple = ultramafic rocks; blues = plutonic rocks (gabbros, dolerites); greens = fore‐arc basalt; yellow‐greens = boninites, and yellow‐orange = andesites. Figures 1a and 1b were created with GeoMapApp. Figure 1c is from Reagan et al. (2015).

The Izu‐Bonin‐Mariana (IBM) system is a convergent plate boundary between the subducting Pacific Plate and the upper Philippine Sea Plate, which currently stretches over 3,000 km from the Izu Peninsula (Japan) to Palau (Figure 1; Stern et al., 2003; Wu et al., 2016). The oldest terranes in the IBM system lie along its periphery and are Cretaceous in age, including the remnant island arcs and ocean islands making up the Oki‐Daito and Daito Ridges and the Amami Plateau (Hickey‐Vargas, 1998, 2005; Ishizuka, Taylor, et al., 2011) in the northern West Philippine Basin, the Huatung Basin abutting Taiwan on the west side of the West Philippine Basin (Hickey‐Vargas et al., 2008), and the Palau Basin at southern end of the Philippine plate (Taylor & Goodliffe, 2004). The oldest back‐arc basin is the West Philippine Basin, which opened between approximately 55–52 and 30 Ma (Deschamps & Lallemand, 2002; Ishizuka et al., 2013; Ishizuka, Tani, et al., 2011; Savov et al., 2006).

Subduction in the IBM system began circa 52 Ma (Ishizuka, Taylor, et al., 2011; Reagan et al., 2013). FAB to boninitic crust generated by this event is found along the much of the IBM fore arc, and similar fore‐arc crust is documented to the south in Tonga (e.g., Meffre et al., 2012), evidence that the subduction initiation event might have spanned much of the western Pacific (Reagan et al., 2013). The tectonic evolution of the IBM from 52 Ma to the present day is complex, involving a myriad of microplates and differential movements. Reconstruction of plate motion and the detail of evolving tectonic scenarios remain an active area of research (e.g., Wu et al., 2016). Generally, as the Philippine Sea Plate grew, it underwent up to 80° of clockwise rotation and moved northward, colliding with SW Japan circa 14–20 Ma (Wu et al., 2016). Back‐arc spreading that began circa 25 Ma created the Parece Vela and Shikoku back‐arc basins (Ishizuka, Tani, et al., 2011), leaving the Kyushu‐Palau ridge remnant arc (Mrozowski & Hayes, 1979; Okino et al., 1998). Back‐arc spreading in the Mariana Trough began separating the active arc from the Mariana Ridge remnant arc at about 6 Ma (e.g., Fryer, 1995). Throughout this long history of subduction and the resulting rear‐arc spreading and arc volcanism, the early arc crust has been largely preserved in the IBM fore arc (e.g., Reagan et al., 2017; Stern et al., 2012). The overall structure of the IBM fore arc includes sedimentary basins bounded by normal and strike‐slip faults, syntectonic pelagic, and volcaniclastic sedimentation with tilting of the lowermost, Oligocene to Early Miocene, sedimentary units (Robertson et al., 2017), and postmagmatic structures including fault zones, slickensides, shear fractures, mineralized veins, and extensional fractures (Christeson et al., 2016).

IODP Expedition 352 was sited in the southern Izu‐Bonin fore arc, east of the Ogasawara (Bonin) island group, and close to the current active trench (Figure 1; Reagan et al., 2015a). These sites were targeted *based* on the results of dredging and submersible dives, which revealed that FABs are exposed deep on the inner wall of the trench, topographically and stratigraphically below boninites (Ishizuka, Taylor, et al., 2011). This location was chosen to sample the oldest volcanic activity associated with subduction initiation and to document the magmatic response to this event as it evolved through time. In contrast, IODP Expedition 351 to the Amami‐Sankaku basin (Site U1438) complemented IODP Expedition 352 by sampling the younger back‐arc side of the subduction initiation event (cf. Ishizuka, Taylor, et al., 2011; Ishizuka et al., 2018), now located west of the Parece‐Vela Basin (Figure 1).

**Figure 2:**
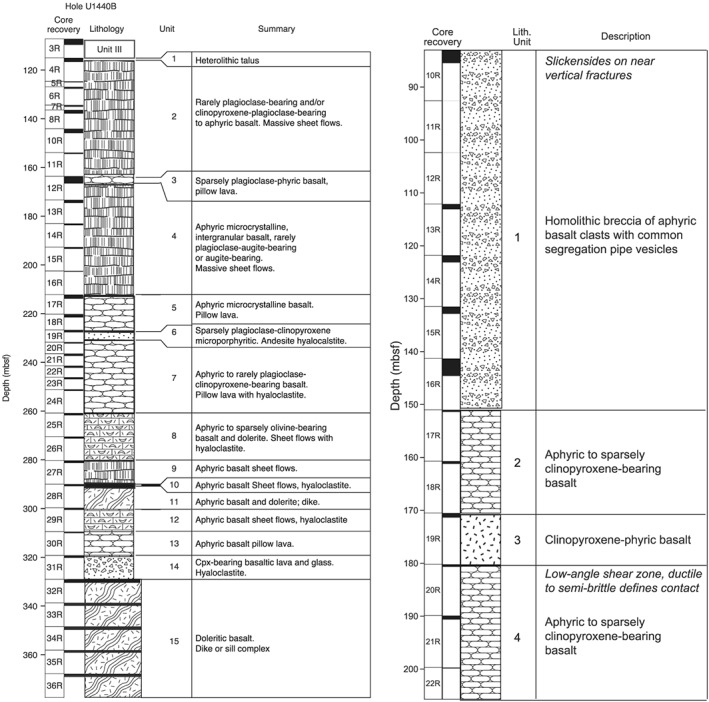
Lithologic columns for Holes (a) U1440B and (b) U1441A, modified from Reagan et al. (2015). Columns show depths (meters below seafloor), core run (*R* = rotary core barrel), recovery for each run (black bars, thickness proportional to recovery), lithologic log, formal unit number, and brief description.

IODP Expedition 352 cored 1.22 km of igneous oceanic crust in the Izu‐Bonin fore arc, including three deep water holes consisting largely of FAB: U1440A (28°26.9890′N, 142°45.2243′E; 4,775‐m water depth), U1440B (28°26.9976'N, 142°45.2244′E; 4,775‐m water depth), and U1441A (28°25.6379′N, 142°43.5390′E; 4,447‐m water depth; Reagan et al., 2015b, 2015c). Over 393 m of volcanic basement was traversed by these three holes: U1440A cored 2.6 m of basement, U1440B cored 268 m of basement, and U1441A cored 123 m of basement (Figure 2; Reagan et al., 2015b, 2015c). Sites U1439 and U1442 were drilled upslope of Sites U1440 and U1441 at water depths of 3,129 and 3,162 m, respectively (Reagan et al., 2015d, 2015e). These sites were targeted to drill from boninites into FAB, but encountered only boninites and related intrusive rocks, leading to the inference that boninitic crust was generated to the west of FAB‐related crust (Reagan et al., 2017).

## 3 Methods

Seventy samples were chosen from Holes U1440A, U1440B, and U1441A. Samples were selected based on apparent freshness and to represent chemical units defined by shipboard chemical analyses. Two to three grams of each sample was reduced to chips and sonified in deionized water repeatedly until the water remained clear then dried and powdered in a tungsten carbide shatterbox. After ignition 1,100 °C, 1.4 g of ignited powder was mixed with 9.8 g of lithium tetraborate flux (1:7 ratio) and fused to form a glass bead for X‐ray fluorescence analysis. The glass beads were analyzed for major elements and Cr with a Panalytical® 2400 sequential X‐ray fluorescence (XRF) spectrometer at Utah State University, calibrated using a suite of 20 international rocks standards (U.S. Geological Survey, Siberian Institute of Geochemistry, and Japan Geological Survey). Count rates were corrected for absorption and secondary fluorescence using fundamental parameters in the Panalytical® SuperQ® software. A split of ~50 mg was dissolved using trace element grade hydrofluoric and distilled trace element grade nitric acid and analyzed with a Perkin‐Elmer 9000 Elan ICP‐MS at Centenary College for the rare earth elements (REEs) and 10 other trace elements. Each sample was spiked with an internal standard of In and Bi to correct for machine drift during analyses. Calibration was done using multielement trace element standards. USGS (United States Geological Survey) and international standards were used to check our calibration curves throughout the analyses. In addition, we have access to data for 40 POOL samples, which were analyzed by ICP‐OES (inductively coupled plasma‐optical emission spetrometry) during the expedition and reanalyzed by XRF afterward (Reagan et al., 2015f). POOL samples were chosen by the Expedition 352 Science Party to encompass the breadth of chemical variability in the recovered igneous material, with the goal of eventually yielding a self‐consistent database of major, trace, and isotopic data on the same homogenized powders. Whole rock major and trace element chemistry is presented in supporting information Table S01 (file 2018GC007731‐Supporting‐Information) and is archived at PANGAEA Data Archiving and Publication (Shervais et al., 2018).

Expedition 352 scientists used a hand‐held portable XRF (pXRF) shipboard to monitor chemical variations in the basalts as part of the core logging process and to define a preliminary chemostratigraphy used to guide subsequent sampling (Ryan et al., 2017). The data from the pXRF were regressed against shore‐based XRF data to determine how well they correlate, and the pXRF concentrations adjusted if needed to remove analytical bias (Cr = Cr_p_*1.28 + 9.8; Zr = Zr_p_*1.38–5.8). No adjustment was made for regressions within 5% of shore‐based XRF values (K_2_O, TiO_2_, and Sr, V). These data are used to supplement shore‐based data for the chemostratigraphy. During shipboard sampling, the pXRF data were used to help select both the POOL and personal samples. This ensured that all chemical groups were sampled for shore‐based studies and that significant chemostratigraphic boundaries were identified.

## 4 Results

### 4.1 Classification and Nomenclature

Table 1 presents a summary of the distinct geochemical lava types found at Sites U1440 and U1141. These geochemical groups are defined in detail below but listed here to facilitate discussion. These include Normal FAB (N‐FAB), Enriched FAB (E‐FAB), Primitive FAB (P‐FAB), Enriched Primitive FAB (EP‐FAB), Depleted FAB (D‐FAB), and Andesite. Andesite has trace element systematics similar to N‐FAB, but at higher concentrations, indicating derivation by fractional crystallization of a common parent magma type. The other geochemical groups have distinct trace element characteristics (supporting information Table S01), which require either different melting histories or distinct source compositions, or both.

**Table 1:** Definition of Chemical Groups, IODP Expedition 352, Based on Rare Earth Element Systematics; Not Normalized

Chemical group	Abbreviation	Ce/Yb	Yb μg/g	Occurrence
Normal FAB	N‐FAB	1.0–2.0	2.0–4.5	U1440: Units 1, 2, 4, 5, 7, 8, 9, 10.11, 12, 13x, and 15a–15e; U1441: Units 2 and 4
Enriched FAB	E‐FAB	2.0–2.6	2.8–3.3	U1440B: Units 13and 14
Primitive FAB	P‐FAB	1.5–2.0	1.7–2.0	U1440B: Unit 3
Enriched Primitive FAB	EP‐FAB	2.0–2.4	1.5–1.8	U1441A: Unit 1
Depleted FAB	D‐FAB	~1.0	1.6	U1441A: Unit 3
Andesite	Andesite	1.0–2.0	>5	U1440B: Unit 6

### 4.2 Volcanic Stratigraphy and Structure.

#### 4.2.1 U1440A

Hole U1440A, a sediment piston core hole, collected two extended core barrel runs at its base, comprising 2.6 m of basement. The basement contains a single igneous unit, a talus breccia of fore‐arc basalt, consisting of angular fragments of microcrystalline basalt. This unit correlates directly with Unit 1 of Hole U1440B, but only the uppermost portion of this unit was penetrated in Hole U1440A. The overlying sedimentary sections are discussed by Robertson et al. (2017) and Kutterolf et al. (2018).

#### 4.2.2 U1440B

In Hole U1440B, located closest to the trench, basement consists of fore‐arc basalt, with ~145 m of lavas and hyaloclastites overlying an ~70‐m‐thick transition zone of lavas, hyaloclastites, and dikes, which in turn overlies a ~55‐m‐thick sheeted dike unit or sill complex at the base (Figure 2a; Reagan et al., 2015b). Igneous rocks in Hole U1440B were divided into 15 chemostratigraphic units by the Expedition 352 science party (Reagan et al., 2015b), based on chemical proxies determined by portable XRF analyses (Ryan et al., 2017), limited whole rock ICP‐OES analyses carried out at sea (Reagan et al., 2015f), and on the physical attributes of the units inferred from core (Reagan et al., 2015b). Below an uppermost unit of volcanic talus (Unit 1), and above the inferred sheeted complex of dolerite sills/dikes (Unit 15), the volcanic section comprises massive sheet flows, pillow lava, and hyaloclastites containing intermingled pillows or sheet flows (Figure 2a). Units 2, 4, and 9 are inferred to comprise massive sheet flows, whereas Units 3, 5, 7, and 13 are inferred to comprise mostly pillow lavas, with one 20 cm‐thick dike in Unit 13 (dike = Unit 13x). Units 6, 8, 10, 12, and 14 are dominantly hyaloclastite with intercalated pillows or sheet flows. Unit 11 is inferred to represent a dike that intrudes a hyaloclastite layer, splitting it into two stratigraphically separate, but chemically identical, units (Units 10 and 12). Unit 15 is the basal dike or sill complex.

Hole U1440B does not display any signs of significant shear localization. There was no evidence that the recovered volcanic sequence was affected by substantial faulting. Multiple sets of fractures without observable shearing were recovered between ~160 and 180 mbsf (Units 2, 3, and 4). These fractures localize alteration producing seams of clays, zeolites, and calcite, with rare sulfides and native copper. At ~358 mbsf, dolerite of Unit 15 was affected by cataclastic deformation and microbrecciation.

#### 4.2.3 U1441A

The stratigraphy of Hole U1441A is relatively simple (Figure 2b; Reagan et al., 2015c), consisting of four igneous chemostratigraphic units defined by the Expedition 352 science party (Reagan et al., 2015c), based on chemical proxies determined by pXRF analyses (Ryan et al., 2017) and on the physical attributes of the units inferred from core: (1) a homolithic breccia of aphyric basalt derived from pillow lava; (2) pillow lava that is chemically and petrographically distinct from Unit 1; (3) a massive sheet flow of highly depleted, augite‐phyric basalt (referred to as *depleted FAB* or *D‐FAB* by Reagan et al., 2015c); and (4) pillow lava that is chemically distinct from the overlying units. The overlying sedimentary sections are discussed by Robertson et al. (2017) and Kutterolf et al. (2018).

Slickensides are abundant between 84 and 88.25 mbsf (uppermost part of Unit 1) and dip steeply subvertically. The general sense of shear is left lateral, strike slip to oblique, reverse also including a left‐lateral component. In the lowermost sections of Hole U1441A, a semiductile to brittle, low‐angle shear zone, defining the boundary between Units 3 and 4, was observed within highly altered domains. The recovered volcanic rocks below and above these units do not show any indication of comparable alteration or deformation. Within the shear zone, shear bands form subparallel sets, indicating top‐down sense of shear. These shear bands are crosscut by subparallel sets of inclined shear fractures, also indicating normal sense of shear.

### 4.3 Petrography and Microstructure

**Figure 3:**
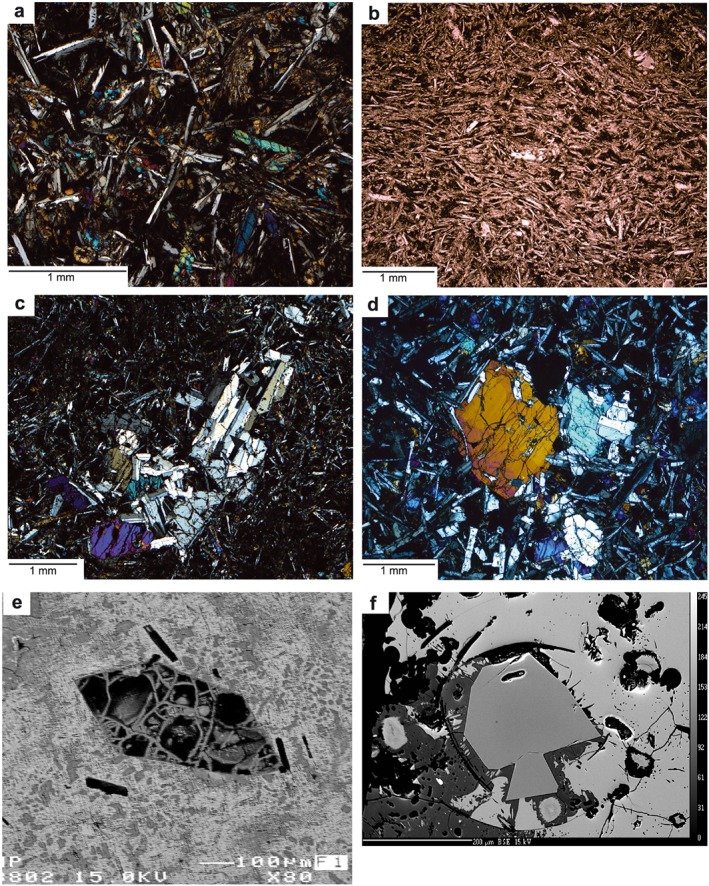
Photomicrographs of FAB. (a) Phyric FAB with subophitic to intersertal textures of augite and plagioclase microphenocrysts; Unit 1 Hole U1440B. (b) FAB with magmatic foliation defined by acicular plagioclase embedded within a microcrystalline matrix, Unit 2 Hole U1440B. (c) Vitrophyric andesite with glomerocrysts of augite and plagioclase in a glassy matrix, Unit 6 Hole U1440B. (d) Doleritic FAB with coarse augite and plagioclase defining subophitic to ophitic textures, Unit 15 Hole U1440B. (e) Euhedral olivine phenocryst in EP‐FAB (Unit 1, U1441A‐10R2‐43/45; TS121), totally altered to chlorite. (f) Unaltered euhedral olivine in P‐FAB glass (Unit 3; U1440B‐12R2‐67/68).

FAB lavas from Expedition 352 form three groups physically: lavas (including both pillow and sheet flow lavas), vitrophyres, and dolerites. The latter represents the dike‐and‐sill complex hypabyssal intrusive rocks in Hole U1440B. Representative photomicrographs are presented in Figure 3, and detailed petrography is reported by Reagan et al. (2015b, 2015c).

#### 4.3.1 Lavas

Pillows and sheet flows are the dominant mode of FAB. They are typically aphyric to sparsely phyric (Figures 3a and 3b); the phyric lavas may be augite bearing, plagioclase bearing, or plagioclase‐augite bearing (≤2% total phenocrysts, typically ≤1‐mm size). The groundmass is microcrystalline with subophitic to intersertal textures, comprising subhedral plagioclase laths ~0.2 mm long (40–50% modal), subhedral blocky augite ≤0.2 mm (15%–25% modal), and a mesostasis of quench crystals ± glass (20%–40% modal; Figure 3a). Vesicles are rare, typically 0%–3% by volume but 5%–15% in rare cases.

#### 4.3.2 Andesite Vitrophyre

Unit 6 in Hole U1440 consists of spherulitic andesite vitrophyres (Figure 3c). These are sparsely microphyric (≤0.8 mm), comprising ~0.5% glomerocrysts of augite and plagioclase set in glass.

#### 4.3.3 Dolerites

Dolerites are found only in Hole U1440B, Unit 15 (Figure 2). They are characterized by seriate grain size distribution, with intergranular, intersertal, subophitic, or hypidiomorphic granular textures (Figure 3d). The seriate textures are fine to medium grained (0.3 to 2.0 mm), with the complete size range preserved in most samples. Plagioclase (50% modal) forms subhedral tabular grains, with rare sieve textures. Augite (30%–40% modal) forms subhedral, blocky‐to‐prismatic grains, commonly ophimottled (augite partially encloses radiating laths of plagioclase, set in matrix of altered glass; Figure 3d). Augite in the dolerites is much lower in Al_2_O_3_ (1.5–2.5 wt%) than augite in most of the lavas. Fe‐Ti oxides are 1%–2% modal and form small (0.1–0.3‐mm) equant interstitial grains. The intersertal patches contain a mesostasis of altered glass that may contain chlorite and quartz.

#### 4.3.4 Primitive Lavas

Primitive lavas, that is, those with high MgO contents, are characterized by rare olivine phenocrysts (Figures 3e and 3f). Where fresh glass is preserved, olivine may also survive unaltered (e.g., Figure 3f), although more commonly, it is altered to chlorite, serpentine, or clays (e.g., Figure 3e).

#### 4.3.5 Microstructure

In general, the magmatic microstructures are isotropic, without indication of a magmatic flow fabric (Figure 3a). Viscous‐plastic fabrics related to magmatic flow are rare and limited to millimeter‐ to centimeter‐wide domains, defined primarily at microscale. The magmatic foliation is mainly defined by the shape‐preferred orientation of acicular feldspar crystals that are embedded within a glassy or microcrystalline matrix (Figure 3b). Glassy materials and fine‐grained FAB generally exhibit circular vesicles. Granular dolerites usually display no shape‐preferred orientation of plagioclase laths. Both basalts and dolerites exhibit a typical radial microstructural arrangement of plagioclase laths attesting to rapid cooling.

### 4.4 Geochemistry

#### 4.4.1 Major Elements

**Figure 4:**
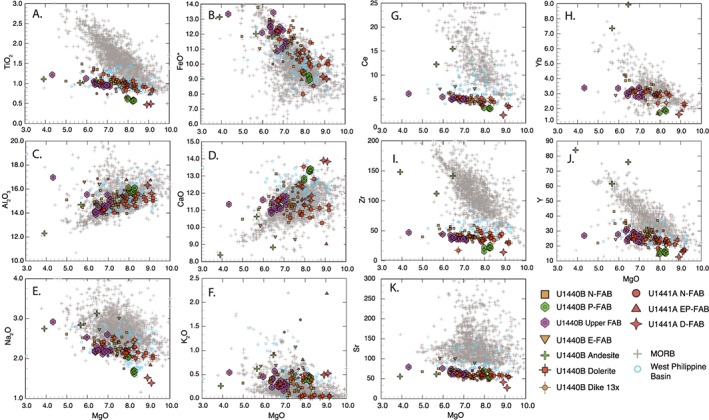
MgO‐variation diagrams for fore‐arc basalts (colored symbols) compared to mid‐ocean ridge basalts (MORBs) and West Philippine Basin spreading centers. MgO weight percent oxide (*x* axis) versus (a) TiO_2_, (b) FeO*, (c) Al_2_O_3_, (d) CaO, (e) Na_2_O, (f) K_2_O, (g) Ce, (h) Yb (μg/g), (i) Zr (μg/g), (j) Y (μg/G), and (k) Sr (μg/g). Expedition 352 FABs are consistently more depleted than any found in the ocean basins. Ocean basin analyses from the PETDB geochemical database. Philippine Sea Plate data from Mattey et al., 1980; Holes 447A, 449, and 450.

Major element geochemistry of FAB is compared with MORBs in MgO‐variation diagrams (Figure 4). FAB and MORB span similar ranges of MgO, Al_2_O_3_, and CaO (Figure 4), but with higher CaO/Al_2_O_3_ ratios (Figure S01A). FABs are consistently lower in TiO_2_ and P_2_O_5_ (Figure S01B) compared to MORB, with Na_2_O at the low end of the MORB field and FeO* at the high end (Figure 4). The range in MgO concentrations (5.5–9.0 wt%) is consistent with limited fractionation, but the high FeO* in the more evolved basalts (up to 14 wt%) implies shallow closed system fractionation to form ferrobasalts (Villiger et al., 2007). Relatively primitive major element compositions (whole rock MgO ≥ 8.0%; mg# ≥60) are found in specific units: in Hole U1440B, Unit 3 and the lowermost dolerites (Units 15d and 15e), and in Hole U1441A, Unit 1 breccia and Unit 3 D‐FAB. The high SiO_2_ supports relatively high degrees of partial melting, as do the high CaO/Al_2_O_3_ ratios (Figures S01C and S01D). The depleted FAB (D‐FAB) of Hole U1441A has higher CaO than either MORB or other FAB, while its TiO_2_ and Na_2_O are noticeably lower (Figure 4).

There is no evidence to suggest significant mobility of most major elements due to secondary alteration. Loss on ignition is generally low (−0.02 to 4.5; average 1.16%), CaO is high, and Na_2_O is low relative to MORB, which is the opposite of what would be expected for low‐temperature alteration. SiO_2_, MgO, and FeO* are all within ranges expected for normal MORB. K_2_O concentrations, however, scatter considerably from 0.04 to 2.0 wt% independently of the concentrations of less mobile elements, which is a hallmark chemical signature of secondary alteration.

#### 4.4.2 Trace Elements

**Figure 5:**
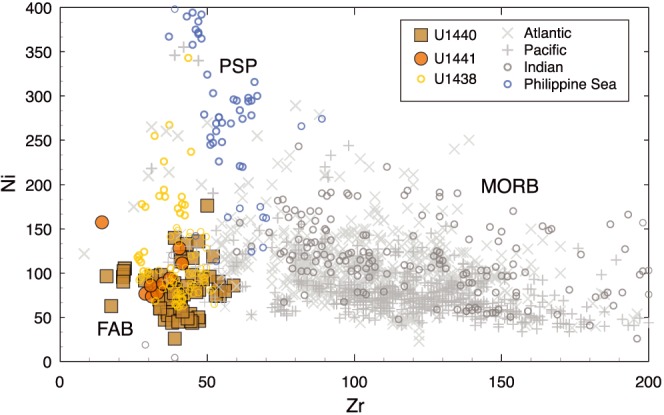
Zr versus Ni in FAB compared to MORB (Atlantic, Pacific, and Indian) and Philippine Sea Plate (PSP) basalts; also shown are depleted basalts from the Amami‐Sankaku back‐arc basin (Expedition 351 Site U1438; Hickey‐Vargas et al., 2018). FABs are strongly depleted in Zr, but also low Ni, as are the Amami‐Sankaku basalts. This is consistent with melting of a depleted source, followed by fractional crystallization. Philippine Sea Plate data from Mattey et al. (1980); Holes 447A, 449, and 450.

High field strength trace elements such as Zr and the light rare earth elements (LREE) define tight linear trends on MgO‐variation diagrams. They are consistently lower in concentration than MORB, with little enrichment over the range in MgO (Figure 4), similar to the minor elements Ti and P. Y and the heavy REE (HREE), such as Yb, overlap MORB, but only at the lowest concentrations (Figure 4). Ni and Zr concentrations are also low relative to MORB, and much lower than basalts from the West Philippine Basin, which have low Zr but at much higher Ni concentrations (Figure 5). Depleted basalts from Expedition 351 (Hole U1438E; Hickey‐Vargas et al., 2018) have similar low Ni relative to Zr (Figure 5). Th/Yb shows no enrichment relative to Nb/Yb compared to the global MORB array and Site U1438, suggesting that there is no discernable enrichment of FAB sources with high‐temperature fluids from a subducting plate (e.g., supporting information Figure S02).

**Figure 6:**
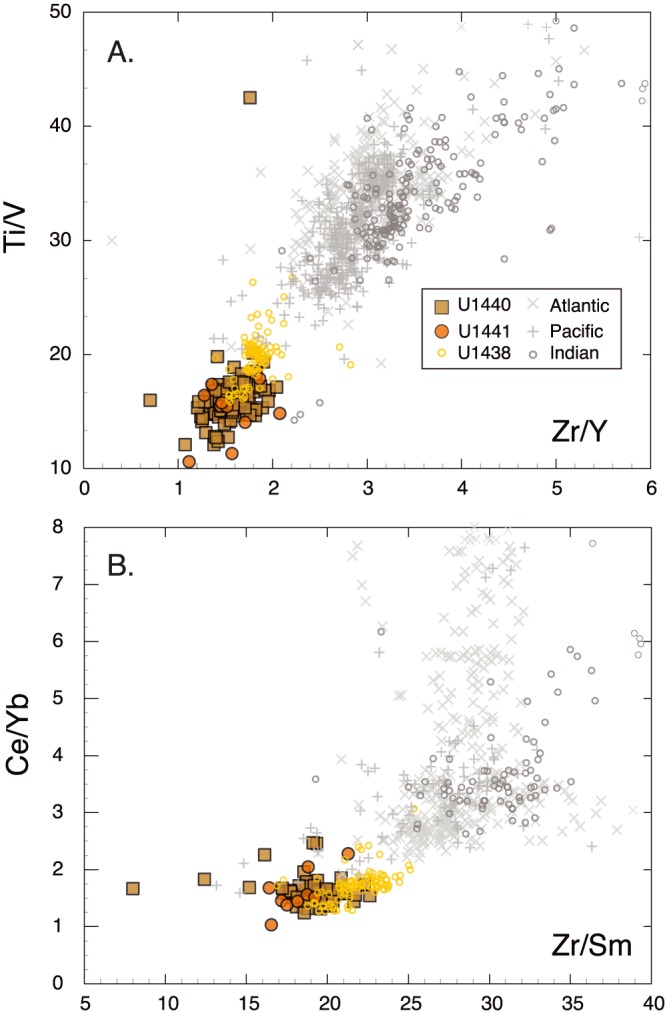
Trace element ratio plots of more incompatible elements relative to less incompatible elements: (a) Zr/Y versus Ti/V and (b) Zr/Sm versus Ce/Yb. Low Zr/Y, Ce/Yb, and Zr/Sm ratios relative to MORB indicate prior melt depletion events; low Ti/V may result either from prior melt depletion events or higher oxygen fugacity during melting, or both. The positive correlation with Ti/V with Zr/Y implies that prior melt depletion is the dominant cause of the low Ti/V ratios.

Ratios of more incompatible to less incompatible elements (e.g., Zr/Y ~1.55, Ce/Yb ~1.61, Ti/V ~10–22, Zr/Sm ~20, and Zr/Hf ~26–30) are lower than MORB (Zr/Y >2.0; Ce/Yb >2.5 largely, Ti/V ~20–50, Zr/Sm ~25–40, and Zr/Hf ~30–50). This is clearly seen in plots of Zr/Y versus Ti/V and Zr/Sm versus Ce/Yb (Figure 6), which also show that depleted basalts from Expedition 351 Site U1438 are similar to FAB in Ce/Yb but have Ti/V, Zr/Y, and Zr/Sm ratios that are intermediate between FAB and MORB. These trends are consistent with either higher degrees of partial melting in FAB compared to MORB or previous melt extraction to form more refractory source regions (see section 6).

**Figure 7:**
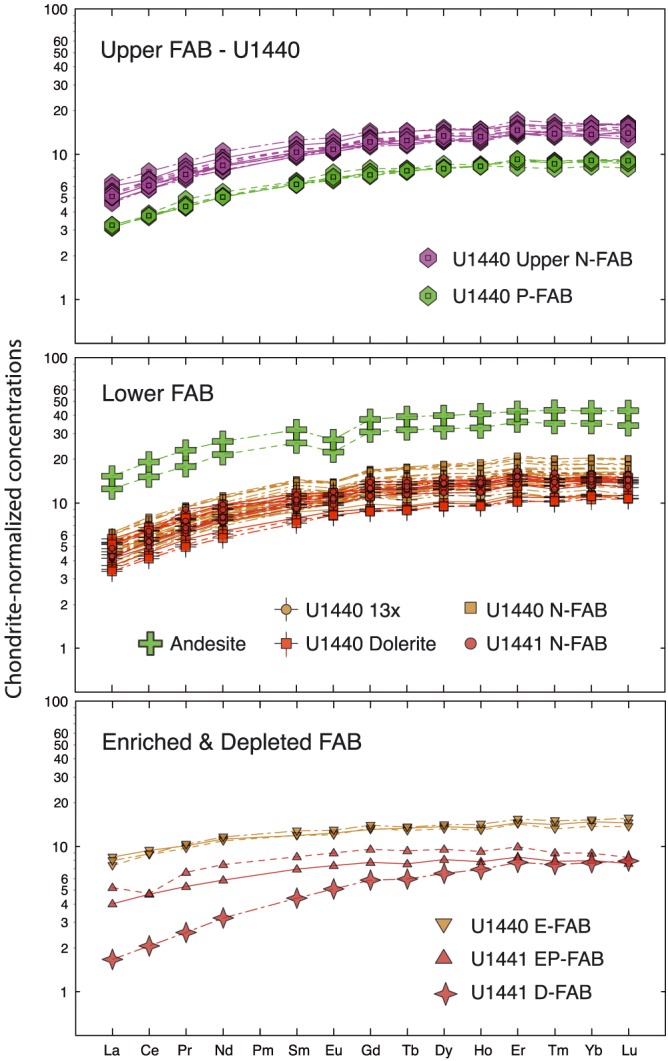
Chondrite‐normalized rare earth element concentrations in fore‐arc basalt (FAB): (a) Upper N‐FAB from Hole U1440, including the Upper P‐FAB unit; (b) N‐FAB from U1440 and U1441, dolerite sills, and the Unit 13X dike from U1440, and andesite; (c) enriched FAB from Holes U1440 and U1441, and D‐FAB from Hole U1441. All samples are LREE depleted, but only the andesites have prominent negative Eu anomalies, indicative of plagioclase fractionation. A few of the more evolved normal FAB have slight negative Eu anomalies. Normalized to the recommended chondrite values of Boynton (1984).

Rare earth element compositions (REE) define five distinct magma types, as seen in chondrite‐normalized REE plots (Figure 7) and summarized in Table 1: (1) Normal FABs (N‐FAB) make up the most voluminous FAB subtype recovered on Expedition 352. N‐FABs are characteristically LREE depleted, with HREE ~4x to 10x chondrite and LREE at 1.5x to 4x chondrite, and have no discernable Eu anomaly except in the most evolved samples. Physically, this group contains all of the dolerites and most lavas. (2) Andesites from Site U1440 Unit 6 have LREE‐depleted abundance patterns similar to those of FAB, but at much higher concentrations, with small negative Eu anomalies, consistent with olivine‐pyroxene‐plagioclase fractionation. (3) Primitive FABs (P‐FAB), found in Hole U1440B Unit 3, are high in MgO and Cr and exceptionally low in TiO_2_ (<0.6 wt%), with REE patterns similar to N‐FAB, but at extremely low REE concentrations (Ce <3.3 μg/g and Yb <2.0 μg/g), setting them apart from N‐FAB. (4) Depleted FAB (D‐FAB) from Hole U1441A Unit 3 is significantly more depleted than other FAB samples, with lower overall concentrations (e.g., Ce ~2x chondrite and Yb ~8x chondrite) and lower LREE/HREE ratios. (5) Enriched FABs (E‐FAB; Hole U1440B Unit 13) are less depleted in LREE than N‐FAB, with overall REE concentrations similar to normal MORB. (6) Enriched Primitive FABs (EP‐FAB; Hole U1441A Unit 1) have REE patterns like E‐FAB, but with extremely low REE concentrations (e.g., Yb < 2 μg/g). P‐FAB (Hole U1440B), D‐FAB, and EP‐FAB (Hole U1441A) all have whole rock Mg# >60, consistent with their low HREE concentrations.

**Figure 8:**
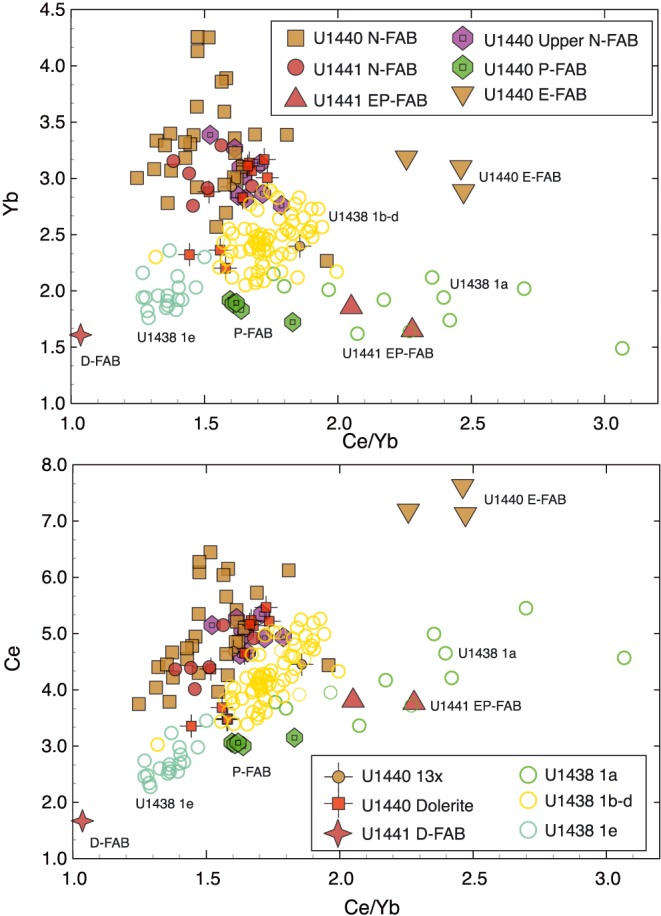
Ce/Yb ratio versus (a) Yb and (b) Ce concentrations in μg/g. N‐FABs have Ce/Yb ratios ~1.3 to 1.8 with Yb > 2.0 μg/g and Ce > 3.5 μg/g. P‐FABs have similar Ce/Yb ratios but with lower Yb (~1.95 μg/g) and Ce (~3 μg/g). E‐FABs have higher Ce/Yb ratios (2.0–2.5) that are similar to MORB (~2.45), but although Site U1440 E‐FAB has MORB‐like concentrations, Site U1441 E‐FAB is strongly depleted, with only ~1.6–1.8‐μg/g Yb and ~3.6–3.8‐μg/g Ce. D‐FAB is strongly depleted in both Ce/Yb ratio (~1.03), Yb (~1.6 μg/g), and Ce (~1.8 μg/g). Basalts of the Amami‐Sankaku basin (Hickey‐Vargas et al., 2018) display a wide range in Ce/Yb ratios but are strongly depleted compared to N‐FAB. See text for discussion.

These magma types are further distinguished in plots of Ce/Yb ratio versus Yb and Ce concentration (Figure 8). N‐FABs are depleted relative to MORB in both REE concentrations and LREE/HREE ratios. E‐FAB and EP‐FAB are characterized by relatively high Ce/Yb ratios (slightly less than normal MORB) at both high (Site U1440) and low (Site U1441) Ce and Yb. In contrast, P‐FAB has Ce/Yb similar to N‐FAB, but at much lower Ce (<3.3 μg/g) and Yb (<2.0 μg/g) concentrations. D‐FAB has lower Ce/Yb, Ce, and Yb than any of the other chemical groups. These differences result in significant differences in melting models (below).

**Figure 9:**
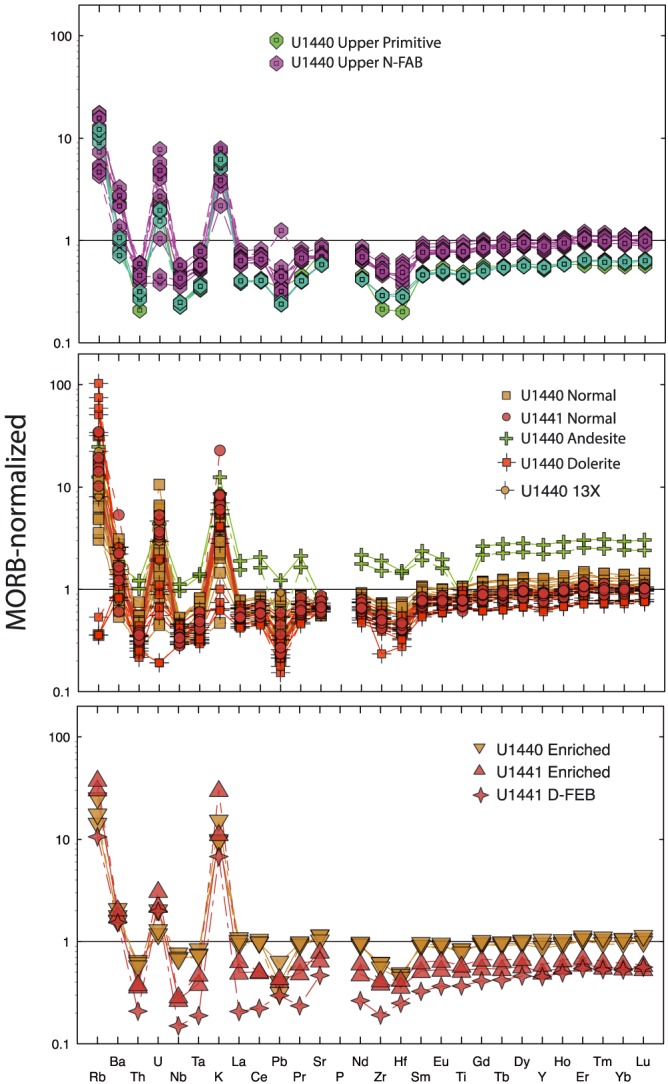
Multielement variation (spider) diagrams, normalized to primitive mantle of McDonough and Sun (1995); same chemical groups and symbols as Figure 8: (a) Upper N‐FAB and P‐FAB from Site U1440; (b) N‐FAB from Sites U1440 and U1441, dolerite sills and the Unit 13x dike from U1440, and andesite; (c) E‐FAB from Site U1440, EP‐FAB from Site U1441, and D‐FAB from Site U1441. All samples show overall depletion trends in the more incompatible elements, with spikes in several fluid‐mobile elements (Rb, Ba, U, and K) that are probably related to low‐temperature alteration. Zr and Hf are deleted relative to adjacent rare earth elements Sm and Eu.

MORB‐normalized multielement (spider) diagrams illustrate the general depletion of FAB in the highly incompatible elements (Figure 9). In detail, however, there are additional deviations from MORB compositions. Zr and Hf are depleted relative to Sm, whereas fluid‐mobile elements (FME: K, Rb, Ba, and U) are commonly enriched relative to MORB or less depleted than expected relative to other more incompatible elements. Most or all of this FME enrichment is likely secondary, documented by the variability of these enrichments in FAB whole rocks, and the lack of similar enrichments in FAB glasses (e.g., Coulthard et al., 2017).

#### 4.4.3 Comparison With Site U1438

Basalts from IODP Expedition 351 (Hole U1438E; Hickey‐Vargas et al., 2018; Ishizuka et al., 2018) are more primitive than most Expedition 352 FAB, with MgO contents as high as 13.7% and averaging 8.6% (i.e., about the same as the maximum MgO content in Expedition 352 FAB). In addition, Site U1438 basalts have lower Ce and Yb at a given Ce/Yb compared with Expedition 352 N‐FAB (Figure 8). However, the most primitive Expedition 352 basalts (P‐FAB, EP‐FAB, and D‐FAB) have still lower concentrations of Ce and Yb. Indeed, Site U1441 D‐FAB has the lowest Ce and Yb concentrations and Ce/Yb ratio (Figure 8) of all basalts from both expeditions. These observations imply that parental magmas for Expedition 352 lavas had lower concentrations of incompatible trace elements compared with Expedition 351 lavas but that Expedition 352 magmas typically underwent more differentiation. Hole U1438E basalts from Unit 1a are the most enriched, with high Ce/Yb ratios and a negative slope in Ce/Yb‐Yb that have been interpreted to represent the effect of significant hydrothermal alteration (Hickey‐Vargas et al., 2018.

### 4.5 Chemostratigraphy

The chemical stratigraphy of volcanic rocks sampled by drill core offers valuable information on the relative timing of chemical variations that relate to both magma supply and crustal processes (e.g., Rhodes & Vollinger, 2004; Jean et al., 2013; Potter et al., 2018; Hickey‐Vargas et al., 2018). Variations in source compositions and melting processes for primitive magmas may be inferred from changes in major element chemistry or incompatible trace element ratios, whereas crustal processes result in open or closed system fractionation trends and mixing arrays (e.g., Shervais et al., 2006).

### 4.6 U1440A

**Figure 10:**
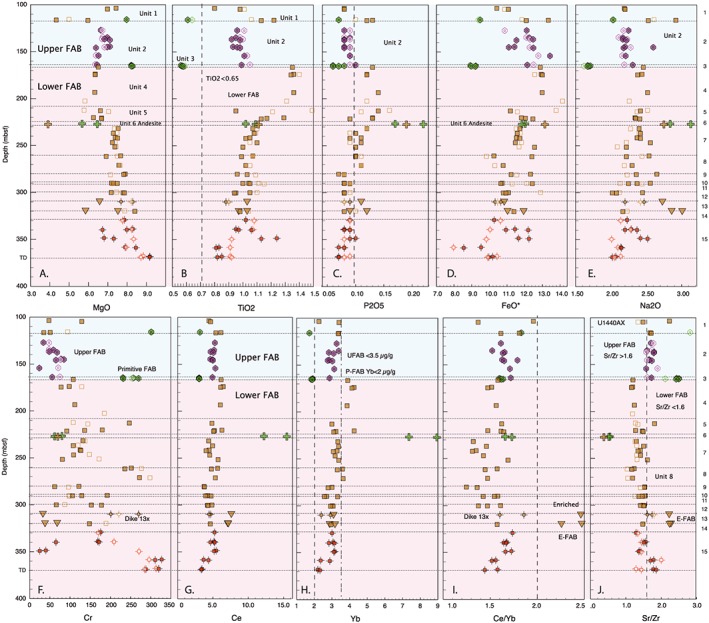
Chemical stratigraphy of Hole U1440A (100–110 mbsf) and U1440B (115–370 mbsf), plotted on same depth scale. (a) MgO wt%, (b) TiO_2_ wt%, (c) P_2_O_5_ wt%, (d) Zr (μg/g), (e) Sr (μg/g), (f) Cr (μg/g), (g) Ce (μg/g), (h) Yb (μg/g), (i) Ce/Yb ratio, and (j) Sr/Zr ratio. Unit numbers listed along the right‐hand axis. See text for discussion. Symbols as in Figure 8, except that open symbols are the POOL samples (Reagan et al., 2015a). See text for discussion.

Hole U1440A contains only a single igneous unit, a talus breccia that correlates with Unit 1 of U1440B (Figure 10). This unit contains a mix of FAB lava types with a wide range in compositions, for example, 5%–8% MgO, 9.5%–13.5% FeO*, and 0.6%–1.2% TiO_2_ (ranges include Unit 1 samples from U1440B).

### 4.7 U1440B

Hole U1440B contains two chemostratigraphic groups: Units 1–3 comprise the *Upper* FAB chemostratigraphic group, whereas the *Lower* FAB chemostratigraphic group comprises Units 4–15 (Figure 10).

The Upper FAB chemostratigraphic group of Hole U1440B comprises Units 1–3 (<166 mbsf depth). Unit 1 (0.52 m thick) is talus breccia with mixed chemical compositions that correlates directly with Unit 1 in Hole U1440A. Unit 2 (48.3 m thick) comprises N‐FAB with intermediate compositions, but with distinctly lower TiO_2_, Zr, Ce, Yb, and Sr/Zr than deeper units (below Unit 3; Figure 10). Unit 3 is extremely thin (2.14 m thick) consisting entirely of P‐FAB with TiO_2_ < 0.58 wt%, MgO >8.0 wt%, and exceptionally low FeO*, Na_2_O, and Yb <2.0 μg/g).

The Lower FAB chemostratigraphic group underlies a break in chemostratigraphic trends at the Unit 3/4 boundary that shift downward to more differentiated compositions (Figure 10) including sharp increases in TiO_2_, P_2_O_5_, Zr, and Yb, as well as a shift in Sr/Zr from >1.6 in the Upper FAB group, to <1.6 in most Lower FAB samples driven by a marked increase in Zr concentration with only a modest increase in Sr beneath the Unit 3/4 boundary.

Units 4 (46.1 m thick) and 5 (14.3 m thick) consist of relatively evolved N‐FAB, with lower MgO (~6.5 wt%) and higher TiO_2_, Zr, Ce, and Yb than shallower units (Figure 10). Unit 6 is only 0.8 m thick (Reagan et al., 2015c). It comprises andesite with variably high SiO_2_ (54–57 wt%), elevated REE concentrations, negative Eu anomalies, low Cr, and high Zr, Hf, and Y concentrations. Units 7 to 12 have intermediate MgO (7–8 wt%), and TiO_2_ (0.9–1.2 wt%), with variable Cr (Figure 10). Unit 11 (12.9 m thick), which was inferred to be a dike by the Shipboard Party, has low Cr relative to the *wall rock* lava units above it (Unit 10; 0.4 m thick) and below it (Unit 12; 6.2 m thick), which are themselves similar in composition.

Unit 13 comprises E‐FAB, crosscut by a 20‐cm‐thick dike of N‐FAB (Dike 13x; Figure 10). When considering most major elements, E‐FABs are similar to N‐FABs (e.g., MgO ~6–7 wt%, TiO_2_ ~1.0 wt%, and FeO* ~11–12 wt%; Figure 10). However, Na_2_O concentrations are higher (2.6–3.0 wt%), and trace element compositions are distinctive. For example, Sr/Zr ratios are higher than all other FAB (>2), driven by high Sr concentrations (>90 μg/g) at relatively constant Zr concentration (40–45 μg/g; Figure 10). Ce/Yb values also are markedly higher for E‐FAB (2.2–2.5), reflecting a flatter, more MORB‐like rare earth element pattern (Figure 8). Unit 14 (10.2 m thick) marks a return to N‐FAB compositions.

Unit 15 (≥40 m thick, from 329.1 mbsf to total depth) was interpreted to represent a sheeted dike/sill complex. Unit 15 includes primitive N‐FAB, with relatively high MgO (>8 wt% in most samples), low TiO_2_ (0.8–1.0 wt% mostly), P_2_O_5_ < 0.1%, Y < 28 μg/g, and Cr = 160–330 μg/g (Figure 10). Unit 15 also contains a more evolved N‐FAB, with low MgO and Cr and higher TiO_2_, P_2_O_5_, and Y.

The overall trend in U1440B is a change from dominantly more primitive deep in the hole to more evolved upsection through Unit 4: MgO and Cr tend to decrease upsection, whereas SiO_2_, TiO_2_, P_2_O_5_, FeO*, Na_2_O, Zr, Ce, and Yb all increase up to the base of Unit 3, where compositions abruptly become more primitive. Key elemental ratios such as Ti/V (~14–20) and Ti/Zr (~160) are remarkably consistent upsection, with the exceptions of Unit 3, and in the andesites where magnetite fractionation resulted in low TiO_2_ concentrations, and thus, low Ti/V and Ti/Zr ratios.

### 4.8 U1441A

**Figure 11:**
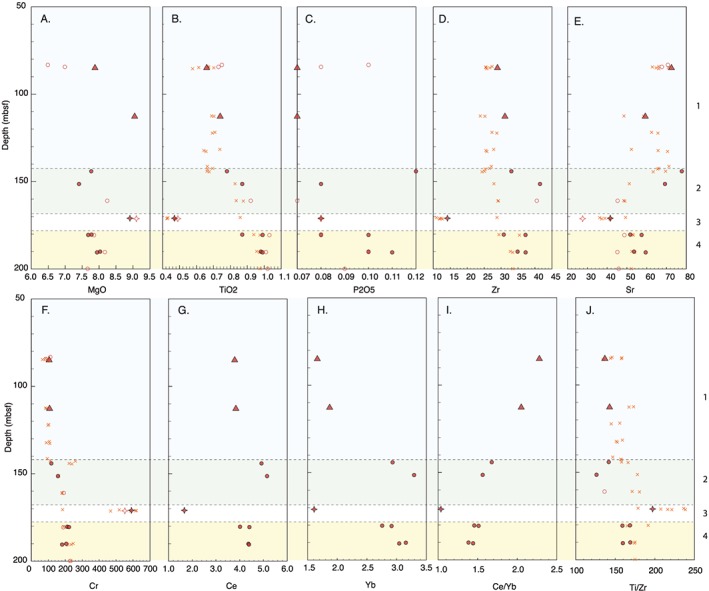
Chemical stratigraphy of Hole U1441A (50–200 mbsf). (a) MgO wt%, (b) TiO_2_ wt%, (c) P_2_O_5_ wt%, (d) Zr (μg/g), (e) Sr (μg/g), (f) Cr (μg/g), (g) Ce (μg/g), (h) Yb (μg/g), (i) Ce/Yb ratio, and (j) Ti/Zr ratio. Unit numbers listed along the right‐hand axis. Symbols as in Figure 8, except that open symbols are the POOL samples (Reagan et al., 2015a) and *x* are pXRF data (Ryan et al., 2017). See text for discussion.

Hole U1441A contains only four units, one of which is D‐FAB (Unit 3), which contrasts with lavas stratigraphically above and below it by having higher MgO and Cr concentrations, and much lower incompatible element concentrations (Figure 11). It should be noted that the composition of D‐FAB cannot be accounted for by accumulation mafic phases, as D‐FAB Unit 3 samples have only about 2% phenocrysts of olivine, plagioclase, and clinopyroxene. Given the limited recovery of Unit 3 (D‐FAB), its unique composition could easily have been missed if not for systematic pXRF scanning of all recovered core during Expedition 352 (Ryan et al., 2017). Like for Hole U1440B, the overall trend for the U1441A N‐FAB is a progressive change from dominantly more primitive deep in the hole to more evolved toward the seafloor. This is shown best by decreasing Cr concentration upsection, from ~200 μg/g to around 100 μg/g.

Of note is the observation that incompatible elements such as TiO_2_, P_2_O_5_, Zr, Ce, and Yb also decrease upsection—the opposite of what would be expected from fractional crystallization or variable degrees of partial melting of a homogeneous source. Key ratios such as Ti/V (~13–18) and Ti/Zr (~120–180) are remarkably constant upsection, apart from the D‐FAB (≤10 and ~200+). The homolithic nature of the Unit 1 breccia is documented by its relatively tight compositional ranges; this unit consists of EP‐FAB (i.e., less depleted in LREE than in N‐FAB), though the overall abundance levels of all REE are very low, with Yb <2 μg/g—similar to HREE concentrations in U1440B Unit 3 P‐FAB. Other incompatible trace element concentrations (e.g., Ti, Sr, V, Hf, and Y) are also lower than N‐FAB. Unit 1 is the most altered in U1441A, with high K_2_O and Rb relative to the deeper Units—most likely as a result of occurrence as a breccia.

## 5 Petrogenetic Modeling

Petrogenetic models address two aspects of FAB origin: (1) partial melting to form the primary magmas and (2) fractionation of these primary magmas to form the observed range of FAB compositions. The partial melting models assume a MORB source composition mantle (commonly referred to as *depleted MORB mantle* or DMM; Salters & Stracke, 2004; Zindler & Hart, 1986). Fractionation models start with the most primitive samples based on their MgO concentrations.

## 6 Fractionation Modeling

**Figure 12:**
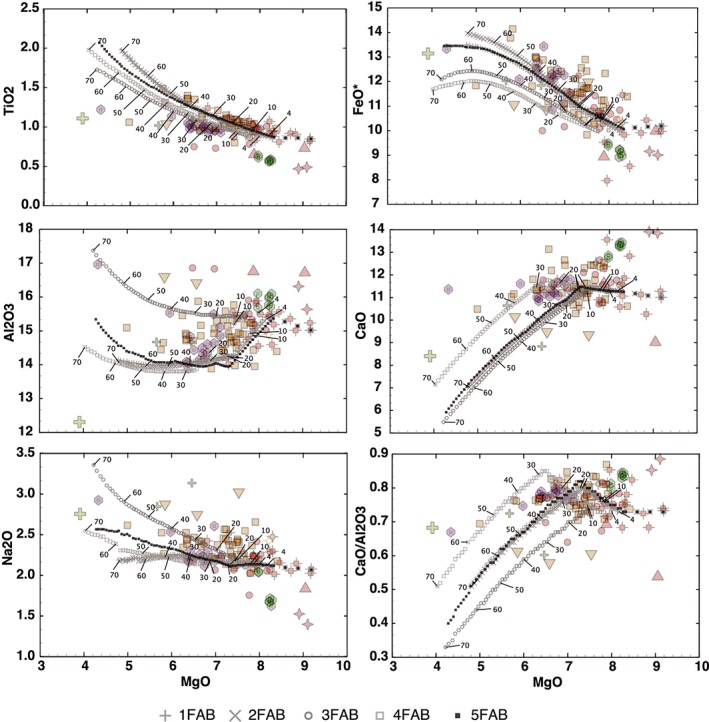
Liquid Lines of Descent (LLD) modeling of FAB fractionation trends from Comagmat (Ariskin, 1999), plotted on MgO variation diagrams with U1440 and U1441 FABs. FAB symbols as in previous figures. Fractionation models 1 through 5 use high‐MgO N‐FAB dolerite from unit 15 as parent. (a) TiO_2_, (b) FeO*, (c) Al_2_O_3_, (d) CaO, (e) Na_2_O, (f) P_2_O_5_. Model 1: Isobaric crystallization at 2 kb, QFM buffer, no water; model 2: Isobaric crystallization at 2 kb, NNO buffer, no water; model 3: Isobaric crystallization at 2 kb, QFM buffer, 0.4 weight % H_2_O; model 4: decompression crystallization at 2 to 0.1 kb, dP/dF = 0.025 kb/mol%, QFM buffer, 0.4 weight % H_2_O; and model 5: isobaric crystallization at 0.2 kb, QFM buffer, 0.2 weight % H_2_O. Symbols as in Figure 8. Tick marks are shown every 10% for models 2FAB, 3FAB, and 4FAB, as well as 4% ticks (near inflection in TiO_2_).

Fractionation modeling of the Lower N‐FAB compositions was carried out using Comagmat petrologic modeling software (Ariskin, 1999). Potential parental magma compositions were chosen based on several factors: high MgO content, low REE concentrations that parallel other FAB, and low incompatible element concentrations. The samples that best meet these criteria are high‐MgO Unit 15e dolerites, which have primitive compositions that could be parental to N‐FAB. D‐FABs have Ce/Yb too low to be parental to N‐FAB, whereas E‐FABs have Ce/Yb, which are too high (Figure 8). P‐FABs of U1440 Unit 3 have appropriate Ce/Yb ratios but very low REE concentrations (Figure 8). As a result, we use Unit 15e high‐MgO dolerite for fractionation modeling here (352‐U1440B‐36R‐1‐W 42/45). Five modeled Liquid Lines of Descent (LLDs) are shown in Figure 12:

- Isobaric fractional crystallization at 200 MPa along FMQ (model 1FAB) under dry conditions;
- Isobaric fractional crystallization at 200 MPa along NNO oxygen buffer (model 2FAB) under dry conditions;
- Isobaric fractional crystallization with 0.4% H_2_O at 200 MPa along FMQ buffer (model 3FAB);
- Polybaric fractional crystallization with 0.4% H_2_O along FMQ buffer (model 4FAB). In the course of crystallization, pressure was continuously decreased from 300 to 20 MPa. Crystallization temperatures were also slightly tuned in order to observe near liquidus (within ±5 °C) olivine + plagioclase cotectic crystallization (temperature corrections +10 °C for olivine and plagioclase) and delay of clinopyroxene crystallization (~15 °C);
- Isobaric fractional crystallization with 0.3% H_2_O at 100 MPa along FMQ buffer (model 5FAB). Crystallization temperatures were also slightly tuned in order to observe near‐liquidus (within ±5 °C) olivine + plagioclase cotectic crystallization (temperature corrections ~10 °C for olivine, +15 °C for plagioclase) and delay of clinopyroxene crystallization (~10 °C).

Unsurprisingly, recovered FAB compositions do not define a single fractionation trend from the same parental magma. However, the scattering of FABs around LLDs (e.g., CaO/Al_2_O_3_, Na_2_O, and FeO) calculated for the range of chosen conditions indicates only modest variability of N‐FAB parental melts (with respect to major elements) or on the range of crystallization conditions, or both. Our best fractionation models (models 4FAB and 5FAB) require ~3%–38% fractional crystallization to reproduce the observed range of compositions, with the bulk of samples falling in the range of 5% to 24% fractionation. Typical model results are progressive crystallization of olivine 1%, olivine + plagioclase 4%–5% (olivine:plagioclase proportions ~30:70), and olivine + plagioclase + augite 17%–20% (olivine:plagioclase:augite proportions ~7:41:52).

Crystallization pressures likely do not exceed 200–300 MPa. Higher pressures would promote earlier clinopyroxene crystallization and would result in an earlier decrease of CaO/Al_2_O_3_ in melts, which is not observed in natural compositions. In fact, low‐pressure and low H_2_O LLD (e.g., 100 MPa, model 5FAB) or low H_2_O polybaric LLD (e.g., from 300 to 20 MPa, model 4FAB) define trends reminiscent of those observed in natural evolved FABs. Isobaric LLD at 200 MPa (1FAB or 2FAB) follows the trend of natural FABs within only 7.5%–9% MgO when melts are driven along olivine + plagioclase cotectics (Figure 12). When clinopyroxene joins the solid phase at 200 MPa, residual liquids start to deviate from natural trend (e.g., CaO and Al_2_O_3_). Dry 200 MPa LLD, however, are closer to natural with respect to FeO contents.

The role of redox conditions cannot be resolved—both FMQ and NNO LLDs (1FAB or 2FAB, respectively) are nearly identical, although FeO contents in calculated residual melts are slightly higher under NNO conditions. The presence of small amount of H_2_O (models 3FABs, 4FAB, and 5FAB) results in delay of plagioclase crystallization, which, in turn, results in slightly higher contents of Al_2_O_3_ and lower contents of FeO in residual melts when compared to dry counterparts (e.g., Danyushevsky, 2001; Husen et al., 2016). Parental melt H_2_O contents should not exceed ~0.4% H_2_O; concentrations higher than 0.4% (model 3FAB) result in a trend of stronger Al_2_O_3_ enrichment, which is not observed in natural FABs.

Unit 15e FAB is our best available candidate to represent an N‐FAB parent melt. However, to reproduce FeO content observed in most evolved FABs from Units 2, 7, and 14, earlier stabilization of plagioclase needs to be invoked. In this case, melt Al_2_O_3_ contents start to decrease at about 8.4% MgO, and calculated LLDs show stronger depletion in this component than is observed in natural N‐FAB (Figure 12). Likewise, the observed range in trace element variations requires several distinct parent magmas that vary in composition from Unit 15e FAB.

### 6.1 Partial Melting

Our partial melting models are based on the assumption that the source of early subduction initiation melts is similar to asthenospheric sources for normal MORBs. This assumption is reasonably robust. However, the high *ε*_Hf_ values reported for many FAB and depleted basalt samples (Reagan et al., 2010; Yogodzinski et al., 2018) suggest that the source is more depleted than MORB source asthenosphere and that this depletion was old and involved residual garnet. As a result, we consider two‐stage melting models and single‐stage models. Melt models are based largely on the MREE‐HREE, which are generally immune to fluid enrichments. We then extend the resulting models to other elements to assess the effect of fluid and/or melt enrichments (e.g., Shervais & Jean, 2012). D‐FAB (352‐U1441A‐19R‐1‐W 44/47) and a P‐FAB sample (U1440B‐36R‐1‐W 42/45) are used to monitor the maximum extents of melting, based on their low REE concentrations and primitive major element characteristics. Less depleted samples may represent smaller degrees of partial melting, enrichment by fractionation, or both.

We use the *Depleted MORB source Mantle* (DMM) of Salters and Stracke (2004) as the starting composition and examine models that involve melting in the spinel lherzolite field only, as well as models that begin melting in the garnet field and then continue in the spinel field. Partition coefficients are those used by Shervais and Jean (2012 *)*. Melt proportions for spinel lherzolite and garnet lherzolite are from Gaetani and Grove (1998) and Walter (1998). In spinel field melting, clinopyroxene is depleted from the source at approximately 25%–28% melting, and the source changes from lherzolite or clinopyroxene‐bearing harzburgite to clinopyroxene‐free harzburgite. None of our models for P‐FAB or D‐FAB reach this extent of melting.

Because D‐FAB and, to a lesser extent, some P‐FAB are more depleted in LREE than MORB, we explored the extraction of small melt fractions (1%–3%) in the garnet field prior to spinel field melting. In our models, the refractory residue of garnet field melting is transformed into spinel facies mineral modes prior to continued melting, using the methods of Shervais and Jean (2012). Our models reproduce normal MORB after ~10% melting in the spinel field only or after 1% garnet field melting followed by 7%–8% spinel field melting. These results are consistent with other estimates of normal MORB formation and provide a baseline against which to compare FAB melt models.

We consider three representative models: (a) spinel field melting, (b) formation of 1% garnet field melt pooled with spinel field melts, and (c) two‐stage melting, with extraction of 1% to 3% garnet field melt prior to a later spinel field melting. No lavas have compositions that are consistent with garnet field‐only melting. We note that in order to achieve the low observed LREE/HREE ratios, these early garnet field melts must separate from the source prior to subsequent spinel field melting. Our modeling does not constrain the timing of the early garnet field melting event.

#### 6.1.1 N‐FAB and U1440B P‐FAB

**Figure 13:**
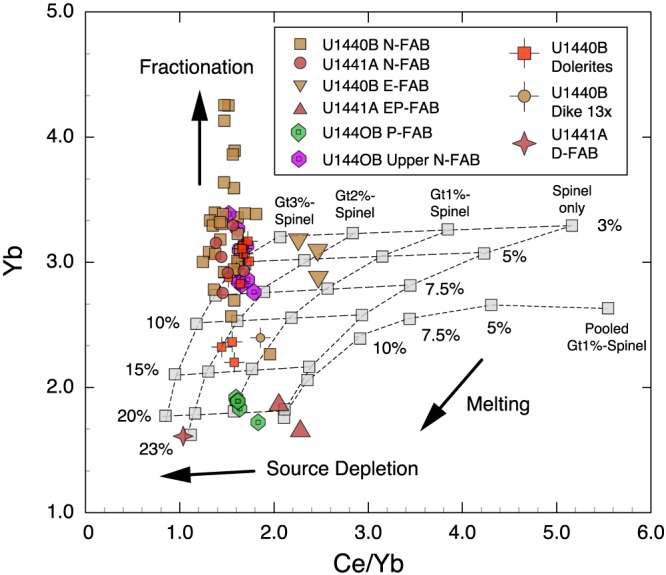
Summary melt model plot of Ce/Yb versus Yb. Melt models are gray boxes, FAB symbols as before. Dotted lines connect melt trends in each model, dashed subhorizontal lines connect equal amounts of spinel field melting. Yb concentration varies with spinel field melt extraction, while Ce/Yb ratio is sensitive to both prior garnet field melt extraction, and total spinel field melt extract. Prior garnet field melt extraction lowers Ce/Yb in subsequent spinel field melts at every melt interval; pooling of garnet field melts with spinel field melts increases Ce/Yb initially, but this effect dissipates as total melt extraction in the spinel field increases. Fractional crystallization will increase Yb without appreciable change in Ce/Yb ratio (vertical arrow). Most N‐FAB fall on a vertical array that projects back to the 2% prior garnet melting model at 7% to 15% spinel field melting. D‐FAB falls on this same array, but at 23% spinel field melting. P‐FABs have Ce/Yb ratios similar to N‐FAB, but with very low Yb concentrations, which require less garnet field melting but at least 20% spinel field melting. U1440 E‐FABs are consistent with 1%–2% prior garnet field melt extraction, followed by 5%–7% spinel field melting. In contrast, U1441 E‐FABs have much lower Yb concentrations but similar high Ce/Yb ratios, requiring at least 20% spinel field melt extraction with or with pooled garnet field melts.

Melt models for N‐FAB and the distinctive U1440B Unit 3 P‐FAB are shown in Figure 13. Spinel field‐only melting cannot reproduce the observed REE patterns, which are too low in LREE and too high in HREE at any melt fraction (Figure S03a). Two‐stage melting with the extraction of 1% garnet field melt prior to 15%–20% spinel field melting reproduces two samples in U1440B, but other FABs are again too low in LREE and too high in HREE at any melt fraction (Figure S03b). Two‐stage models with 2%–3% garnet field melt extraction prior to spinel field melting provides our best match for most N‐FAB, with at least 7%–10% melting in the spinel field; the most primitive dolerites require at least 12%–15% melting in the spinel field after extraction of 2% garnet field melt (Figure S03c). U1440B Unit 3 P‐FABs, which have LREE/HREE similar to N‐FAB, but at lower concentrations, are best modeled by two‐stage melting, with 1% garnet field melt prior to with 20% spinel field melt in melt column (Figure S03). Single‐stage pooled melt models do not reproduce either N‐FAB or P‐FAB (Figure S03d).

#### 6.1.2 E‐FAB, EP‐FAB, and D‐FAB

**Figure 14:**
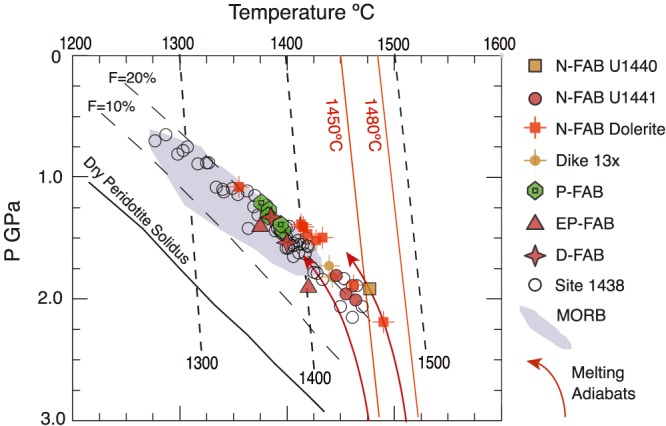
Melting temperature and pressure for the most primitive FAB samples, with MgO >8 wt% and Mg# >60. Temperature and pressure calculated using the method of Lee et al. (2009), using residual mantle compositions of Fo91 and Fe^3+^/Fe_total_ ratios of 0.15. Also shown are P‐T values for Site 1438 depleted basalts from Hickey‐Vargas et al. (2018) and the field for primitive MORB from Lee et al. (2009). Lherzolite solidus (solid black line) and melt fraction isopleths (long dash black lines) are from Katz et al. (2003); Adiabats for mantle potential temperatures of 1,300 °C, 1,400 °C, and 1,500 °C are shown for reference as short dash lines. Red lines are mantle melting adiabats, with potential temperatures of 1,450 °C and 1,480 °C. U1440 and U1441 FABs plot on the low‐pressure, high‐temperature side of the MORB field, near the *F* = 20% melt curve, consistent with relatively shallow melting of slightly refractory mantle, at higher than normal mantle potential temperatures.

Melt models for U1440B E‐FAB, U1441A EP‐FAB, and U1441A D‐FAB are shown in Figure S04. Melts in the spinel field are a fairly close fit for the EP‐FAB at 20% melting; spinel‐only melting cannot match either E‐FAB or D‐FAB (Figure S04a). One E*‐*FAB sample is matched reasonably well by two‐stage melting with 1% garnet field melt removed prior to ~7% spinel field melting (Figure S04b), but the best match for the other E‐FAB samples is provided by two‐stage melting with 2% garnet field melt removed prior to 3%–5% spinel field melting (Figure S04c). D‐FAB is best reproduced by two‐stage melting, with 2% garnet field melt removed prior to 23% spinel field melting (Figure 14c). In contrast, U1441A EP‐FAB may be reproduced by a pooled melt model, with 1% garnet field melt pooled with ~20% spinel field melt (Figure S04d).

#### 6.1.3 Comparison of Partial Melting Models

These partial melt models can be compared using a plot of Ce/Yb ratio versus Yb concentration. Yb concentration is a function of the extent of partial melting and the presence or absence of residual garnet, whereas Ce/Yb varies with the residual assemblage and the extent of melting. In contrast, fractional crystallization will increase Yb concentration without significantly changing the Ce/Yb (Figure 13). Melt model results are shown as gray boxes, with extent of melting labeled and indicated by subhorizontal lines. Ce/Yb ratios and Yb concentration both decrease with increasing melt fraction, but Ce/Yb also decreases with increasing garnet field melting, unless the garnet field melts are pooled with the later spinel field melts. As the extent of partial melting increases, the pooled garnet + spinel field melts approach the composition of spinel‐only melting (Figure 13).

N‐FABs form a near‐vertical array (consistent with fractional crystallization) that projects back to melting models with a minimum of 10% to 15% melting after 1% garnet field melt extraction or a minimum of 7% to 10% melting after 2% garnet field melt extraction (Figure 13). Our N‐FAB parent magma (Unit 15e dolerite) is more refractory, requiring at least 12%–15% spinel field melt after extraction of 2% garnet field melt. These melt models overlap with the extent of melting proposed for MORB, but the prior extraction of garnet field melt results in lower Ce/Yb. U1440B E‐FAB samples cluster near 5%–7.5% melt after 2% garnet field melt extraction, whereas U1441A EP‐FAB has much lower Yb and requires a pooled melt model with 1% garnet +20% spinel field melting. D‐FAB requires 23% melting after 2% garnet field melt extraction (Figure 13). The high degrees of melting calculated for D‐FAB may reflect a larger extent of melt extraction during the first stage of melting (garnet + spinel field melting), rather than extremely high melt fractions during stage two melting.

Multielement diagrams that compare the extended melt models to observed data are shown in Figure S05. Select melt models chosen to reflect best fits to the REE data (shown as gray symbols) are extended to a wider range of elements. In general, the fluid mobile elements K, Rb, Ba, U, and Pb occur in higher concentrations than predicted by the melt models. However, the degree of enrichment of these elements varies dramatically from sample to sample, and irregularly from element to element (Figure S05), which is most consistent with their addition during low‐temperature alteration (K was not included in the melt models but has similarly high and variable concentrations compared with expected high‐degree melts from depleted mantle).

### 6.2 Thermobarometry

Estimates of the pressure and temperature of the magma source were calculated using the method of Lee et al. (2009) on the basis of major element composition of the FAB and, in particular, the activity of silica. The calculations were performed for the least evolved FAB samples (i.e., samples with >8% MgO; supporting information Table S02). We used our constraints from fractional crystallization models for olivine‐saturated melts and tested scenarios encompassing both 0% and 0.5% H_2_O in the primary magma. We explored Fe^3+^/Fe‐total ratios of 0.10 and 0.15 and varied the Mg#s of the assumed residual mantle from 90 to 91. Regardless of chosen conditions, equilibration temperatures are slightly high (~1400 °C) and pressures low (1.4–1.6 GPa or 46–53 km depth) relative to most MORB and to primitive arc lavas (e.g., Perrin et al., 2016). There is negligible difference in results between the anhydrous and 0.5 wt% H_2_O cases. Residual mantle composition asserts a greater effect, with higher Mg# (i.e., a more depleted source) in the residual mantle (Mg# = 91) requiring temperatures about 46 °C higher than less refractory mantle (Mg# = 90) and pressures about 0.32 GPa higher (or about 10 km deeper). However, these P and T variations are on the order of the estimated uncertainty of the thermobarometer (±30–50 °C and ±0.2 GPa; Lee et al., 2009). Indeed, the majority of the conditions explored for FAB equilibration produce P and T results within the uncertainty of the thermobarometric calibration.

We assume a residual mantle olivine composition of ~Fo91 based on Mariana Trench peridotites recovered by dredge and submersible (Michibayashi et al., 2007) and the high degrees of melting implied for primitive FAB magmas. Fe^3+^/Fe ratios are assumed to be 0.15, based on oxygen fugacity studies of Brounce et al. (2015). These results are shown in Figure 14, along with results for Site U1438 from Hickey‐Vargas et al. (2018). The MORB array from Lee et al. (2009) assumes less depleted residual mantle olivine compositions of Fo90, in keeping with the less depleted incompatible trace element compositions of MORB versus FAB. Most FAB parental melts lie along the low‐pressure margin of the MORB array, coincident with the 20% melting curve of Katz et al. (2003), with potential temperatures of ~1,380 °C to 1,480 °C. In detail, N‐FAB samples have the highest potential temperatures (>1,400 °C) at relatively low pressures, while P‐FAB and D‐FAB have lower potential temperatures (≤1,400 °C) at slighter lower pressures (Figure 14). In contrast, U1441 EP‐FAB formed at relatively high pressures relative to temperature, with mantle potential temperatures of about 1,400 °C (Figure 14).

## 7 Discussion

*Fore‐arc basalts* (FABs) were first described and named by Reagan et al. (2010) based on samples recovered by submersible dives in the IBM fore arc from outcrops that were trenchward and deeper than those for boninite. The occurrence of similar suprasubduction zone tholeiitic basalts that predate boninite volcanism was also inferred by Shervais (2001), based on ophiolitic crustal sections with lower crustal plutonic sections that document boninite‐related ultramafic rocks (wehrlites, pyroxenites, and dunites) that intrude and crosscut older, MORB‐like cumulate gabbros. Our current understanding of the relationship between FAB and subduction initiation is that at the time FAB formed, there was no existing island arc: a subduction‐related island arc does not start to form until several million years after subduction initiation, and it is built on a substrate of the recently formed FAB and boninite oceanic crust.

FABs are generally considered to be MORB‐like decompression melts with little or no involvement of subducted fluids in their genesis (e.g., Reagan et al., 2010). However, as can be seen in Figure 3, there are significant differences between FAB and MORB from the Atlantic, the Pacific, and the Indian Oceans. Although they span ranges in MgO (5–9 wt%), FeO* (8–14 wt%), and CaO (10–13 wt%) that are similar to MORB, FABs are characteristically lower in TiO_2_, P_2_O_5_, Zr, and the LREE than MORB from any ocean basin. Diagnostic ratios such as Zr/Y (~1–2.2) and Ti/V (~10–22) also are consistently lower than MORB (Zr/Y >2 and Ti/V >20). Thus, FABs from the IBM arc system either formed by processes that differ from normal MORB or are derived from mantle that is more refractory than DMM.

The question as to whether FABs contain a subduction‐related component is difficult to address with whole rock geochemical data. Many of the elements that would usefully herald a contribution from subduction‐derived fluids are also those most prone to elevation due to seafloor alteration (e.g., Ba, Rb, Cs, U, K, Sr, and Pb). Definitive identification for subduction‐related enrichment in FAB will be addressed in upcoming work on fresh glasses recovered from Expedition 352 (see Coulthard et al., 2017, for a preliminary discussion of glass compositions).

### 7.1 Axial Versus Off‐Axis Volcanism

The distinct break in chemostratigraphic trends in Hole U1440B between Lower FAB units (4–15) and Upper FAB units (1–3) is interpreted here to represent a break between lavas erupted at an axial spreading center (Lower FAB) and those erupted off axis (Upper FAB). The Lower FAB axial lavas display upward fractionation trends to lower MgO and higher FeO*, TiO_2_, and P_2_O_5_, which abruptly reverse to more primitive values in the Upper FAB units. The upward fractionation trends observed in the Lower FAB lavas imply processing in an axial magma system, where magma recharge is buffered by remnants of stagnant magma. In addition, the aphyric nature of the lavas is also consistent with the presence of a melt lens, allowing for efficient segregation of crystals by density separation (e.g., Lange et al., 2013). In contrast, the lowermost Upper FAB unit (Unit 3: P‐FAB with ~8.3% MgO) is among the most primitive encountered in Hole U1440B, consistent with the introduction of a new primitive magma batch that does not intercept an axial magma chamber. The overlying lavas of Unit 2 are much lower in MgO (~6.4%–7.1% MgO) but also have with low TiO_2_ concentrations and high Sr/Zr relative to Lower FAB lavas of Unit 4 (~1% TiO_2_ versus 1.3% TiO_2_), showing that the Upper FAB lavas must have a distinct source compared to those below. The variable differentiation of Upper FAB lavas implies a more complex magma storage system like those found near propagating ridge tips, devals, and off‐axis eruption sites (e.g., Reynolds et al., 1992; Sims et al., 2003). We thus consider eruptions of Upper FAB to be off axis.

The Lower FAB fractionation trend does not include Unit 13 E‐FAB, which represents a distinct magma batch erupted prior to most N‐FAB volcanism. The early intercalation of E‐FAB lava with more common N‐FAB immediately above the dike complex suggests that these lavas erupted prior to formation of a stable melt lens. The Unit 6 andesite indicates a period of in situ fractionation without recharge, followed by Units 4–5, with compositions that are less evolved than andesite. We interpret this as consistent with a semistable melt lens that waxes and wanes in size prior to its demise as the crust moves away from the spreading axis, and off‐axis magmatism prevails (i.e., the Upper FAB lavas).

### 7.2 Melt Models

Our models generally require two distinct melting events, with the first event depleting the mantle source region relative to DMM and the second event producing the FAB compositions we observe. Additionally, our melting models show that most FABs are the product of somewhat higher degrees of partial melting compared to MORB, from a more refractory source. Melting must have begun in the garnet field and continued into the spinel field, assuming a standard DMM composition as the starting point. Our two‐stage melting models require 1%–3% garnet field melt extracted prior to an additional >7%–15% melt in the spinel field for N‐FAB or 23% additional melt for D‐FAB. The requirement for 1%–3% garnet field melting that is extracted from the source prior to spinel field melting is driven by the low LREE/HREE ratios of most FAB. D‐FAB requires at least 2% prior melting in the garnet field, followed by 23% melting in the spinel field (however, we cannot discount formation of D‐FAB from a more refractory source than N‐FAB, e.g., by an early phase of garnet + spinel field melting). These degrees of melting are comparable to those needed for some boninite melts, albeit with additional prior depletion (Pearce & Robinson*,* 2010), and are unusually high for simple decompression melts of MORB source asthenosphere. Thus, the high degrees of melting inferred from these melt models imply a higher mantle potential temperature or greater prior depletion than found under MORB‐generating spreading ridges.

A two‐stage melting model is further supported by Hf‐Nd isotope systematics, which require an older melting event not recorded in normal DMM (Reagan et al., 2010; Yogodzinski et al., 2018) that predates Eocene subduction initiation in the IBM arc. The near‐pervasive requirement for early garnet field melting, along with low Na_8_ and high Fe_8_, implies a deep source with a longer melt column, leading to higher degrees of melting than most mid‐ocean ridge segments (e.g., Klein & Langmuir, 1987). However, the garnet field melts had to separate from their source before spinel field melting began, so melting could not represent a single deep‐seated melt column. In addition, the isotope systematics show that the deep garnet field melting may be older than the Eocene age of subduction initiation (Reagan et al., 2010; Yogodzinski et al., 2018).

### 7.3 Tectonic Setting

Sites U1440 and U1441 FAB are distinct from both Pacific MORB and Indian Ocean MORB in major and trace element systematics, and their origin is inconsistent with derivation from the same mantle sources as either of those MORB suites. They are most similar to basalts of the West Philippine Basin (e.g., Ocean Drilling Program [ODP] Site 447; e.g., Mattey et al., 1980) in terms of SiO_2_, FeO*, CaO, Al_2_O_3_, and Na_2_O, but they also exhibit significant differences (e.g., lower TiO_2_, Sr, Zr, La, Ce, and Ti/V). Compared to basalts from the Amami Sankaku Basin (IODP Expedition 351 Hole U1438E; Arculus et al., 2015; Hickey‐Vargas et al., 2018), IODP Expedition 352 FABs are lower in Sc, Sr, Ti/V, Zr/Y, and Zr/Sm, with similar SiO_2_, FeO*, CaO, Al_2_O_3_, and CaO.

Wu et al. (2016) present a detailed tectonic evaluation of paleoplates in the western Pacific‐Philippine Sea region based on the reconstruction of subducted slabs from tomographic images. According to their preferred model, the Philippine Sea Plate nucleated adjacent to the Manus plume prior to 52 Ma as proposed by MacPherson and Hall (2001). Subduction initiation along the margin of the nascent Philippine Sea Plate circa 52 Ma tapped mantle with an Indian Ocean affinity, whose trace element composition reflected a significantly older episode of small volume melt extraction in the garnet field, potentially leading to higher Hf isotope ratios (e.g., Reagan et al., 2010; Yogodzinski et al., 2018). Expedition 352 FABs were sourced largely from this previously depleted mantle by relatively large fractions of melt extraction in the spinel field. The high melt fractions derived from shallow depths in previously depleted mantle suggest an abnormally hot mantle source region beneath the spreading axis. This is supported by thermobarometry calculations on the least evolved FAB samples, which document relatively high temperatures (~1,400 °C) at low pressures (1.4–1.6 GPa or 46–53‐km depth) relative to most MORB and to primitive arc lavas (e.g., Perrin et al., 2016).

The Manus plume may have played a role in this process by inducing the upflow of deeper, hotter mantle along the West Philippine Ridge, driving high degrees of partial melting of depleted Indian lithosphere or plume‐residue mantle during the early stages of subduction (Ishizuka et al., 2013; Macpherson & Hall, 2001). The Manus plume is currently manifest via helium and oxygen isotopic anomalies in the Manus basin (MacPherson et al., 1998, 2000; Sinton et al., 2003). However, at the time of subduction initiation circa 50–52 Ma, the Manus plume is inferred to have formed an ocean plateau that was fractured by subsequent Philippine Sea spreading to produce the current Oki‐Daito Ridge, Minami‐Daito Basin, Urdaneta Plateau, and Benham Rise (Ishizuka et al., 2013; MacPherson & Hall, 2001).

New radiometric age data show that FABs throughout the IBM fore arc are circa 52 Ma in age and that boninite volcanism began before 51.3 Ma (Reagan et al., 2019). Thus, the transition from fore‐arc spreading to the formation of the nascent Bonin Ridge embryonic arc occurred quite rapidly. Although the oldest Site U1438 basalts have compositions similar to those of FAB from the Bonin fore arc, radiometric dating reveals that they are circa 50 Ma (^40^Ar/^39^Ar age of 49.9 ± 0.5 Ma renormalized to Fish Canyon Sanidine flux monitor of 28.201; Ishizuka et al., 2018). This indicates that they formed not by subduction initiation spreading but most probably by extension within or behind the Bonin Ridge embryonic arc (Reagan et al., 2019). The compositional similarity between the basalt from Site U1438 and Sites U1440 and U1441 FAB thus appears to reflect the depletion history of their common source region, rather than a common tectonic setting.

### 7.4 Subduction Initiation and Suprasubduction Zone (SSZ) Ophiolites

It now seems well established that many SSZ ophiolites form during subduction initiation, in which slab rollback drives early decompression melting to form FAB, and later fluid‐flux melting forms boninites and related rocks, before the establishment of stable steady state, downdip subduction (e.g., Metcalf & Shervais, 2008; Pearce et al., 1984; Reagan et al., 2010; Shervais, 2001). While a full comparison with ophiolites is beyond the scope of this paper, we can make some observations on the IBM system as a paradigm for ophiolite formation.

Our data show that the extent of melting in Izu‐Bonin fore‐arc FAB is higher than normal MORB and requires an unusually depleted source (e.g., Yogodzinski et al., 2018) that was remelted during FAB formation. Oceanic crust of the West Philippine Basin began forming at approximately the time of subduction initiation in the IBM system (e.g., Ishizuka et al., 2018; Ishizuka, Taylor, et al., 2011; Savov et al., 2006). If the preferred model of Wu et al. (2016) is correct, spreading in the West Philippine Basin may be linked to the Manus hotspot, and subduction initiation may have occurred in response to interactions between the Manus hot spot plume and the large offset transform fault that formed the western margin of the Pacific Plate (e.g., MacPherson & Hall, 2001). A similar model has been proposed for the Caribbean plateau and the central America subduction zone (Gerya et al., 2015; Stern & Gerya, 2017; Whattam & Stern, 2015). If so, then the presence of a mantle plume could explain the higher than normal potential temperatures, and some of the extreme depletion of the mantle source could have resulted from decompression melting within the plume. This scenario does need to be reconciled with the occurrence of FAB followed by boninite at a similar time along a front stretching from the Izu‐Bonin region in the north to Guam in the south (e.g., Ishizuka, Taylor, et al., 2011; Reagan et al., 2013).

One implication of our two‐stage melting model is that IODP Expedition 352 FAB may be compositionally unique and not representative of subduction initiation in locations that are characterized by *normal* MORB source mantle. This means that the first subduction initiation melts in other locations are likely to more closely resemble MORB than IBM FAB. This observation is supported by the preboninite lavas and intrusives in many SSZ ophiolites, which are less depleted than IODP 352 FAB and closer in composition to normal MORB (e.g., Godard et al., 2006; MacLeod et al., 2013). A similar conclusion was reached by Yogodzinski et al. (2018) for the depleted U1438 basalts.

## 8 Conclusions

This paper presents the first study of stratigraphically in situ fore‐arc basalt from the earliest stages of the IBM subduction system. This rich data set was enabled by real‐time shipboard chemostratigraphy (Ryan et al., 2017) allowing us to capture the full chemical diversity of FAB recovered. One of the major observations to result from this work is the wide compositional diversity, which reflects variations in source compositions, variations in the extent of partial melting, and subsequent fractional crystallization.

Although IBM FABs are broadly similar to MORB in major element composition (e.g., MgO, FeO*, CaO, and Al_2_O_3_), they exhibit a number of significant distinguishing geochemical characteristics, which include lower concentrations of TiO_2_, Na, Zr, Sr, and the LREE and lower Ce/Yb, Ti/V, Zr/Y, and Zr/Sm ratios. These differences require that FAB form by higher degrees of partial melting than normal MORB. Our melting models also require a two‐stage melting history, with small fractions of garnet field melts extracted sometime prior to later, high fraction spinel field melting. Published Hf‐Nd isotopic studies (e.g., Reagan et al., 2010; Yogodzinski et al., 2018) are consistent with these models, in that the elevated Hf‐Nd ratios require that the early melt extraction event significantly predates the later, shallow event. Thermobarometic calculations imply the last equilibration of FAB melts at shallow pressures and elevated mantle potential temperatures, consistent with decompression melting during the earliest stages of subduction initiation, and additional thermal input from a deeper source, for example, possibly the Manus hot spot.

Understanding the origin of FABs, which are the first products of subduction initiation, is key to evaluating models of subduction initiation in the IBM and more widely. IBM FABs suggest both prior source depletion and high potential temperatures; in this, they may differ from FAB in other subduction initiation settings, which may be more similar to MORB.

## Supporting information



Supporting Information S1

Data Set S1

Data Set S2
