# Supplementary material for: Magmatic Response to Subduction Initiation: Part 1. Fore‐arc Basalts of the Izu‐Bonin Arc From IODP Expedition 352
Source: Geochem Geophys Geosyst. 2019 Jan 16;20(1):314–38. doi: 10.1029/2018GC007731 (PMC6392113; doi:10.1029/2018GC007731)
Supplement: Supplementary file 1 — Supporting Information S1 [file GGGE-20-314-s001.docx]

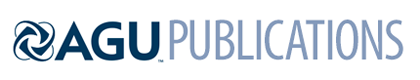


*Geochemistry, Geophysics, Geosystems*

Supporting Information for

**Magmatic Response to Subduction Initiation, Part I: Forearc basalts of the Izu-Bonin Arc from IODP Expedition 352**

John W. Shervais^1^, Mark Reagan^2^, Emily Haugen^1§^, Renat Almeev^3^, Julian Pearce^4^, Julie Prytulak^5^, Jeffrey G. Ryan^6^, Scott Whattam^7^, Marguerite Godard^8^, Timothy Chapman^9^, Hongyan Li^10^, Walter Kurz^11^, Wendy R. Nelson^12^, Daniel Heaton^13^, Maria Kirchenbaur^14^, Kenji Shimizu^15^, Tetsuya Sakuyama^16^, Yibing Li^17^, Scott K. Vetter^18^

^1^Department of Geology, Utah State University, Logan, UT, 84322-4505 USA [*john.shervais@usu.edu*](mailto:john.shervais@usu.edu)*,* [*ehaugen3@gmail.com*](mailto:ehaugen3@gmail.com)*;* ^2^Department of Earth and Environmental Science, University of Iowa, Iowa City, IA, USA [*mark-reagan@uiowa.edu*](mailto:mark-reagan@uiowa.edu)*:* ^3^Leibniz Universität Hannover, Institut für Mineralogie, Callinstrasse 3, D-30167, Hannover, Germany [*r.almeev@mineralogie.uni-hannover.de*](mailto:r.almeev@mineralogie.uni-hannover.de)*;* ^4^School of Earth & Ocean Sciences, Cardiff University, Cardiff, UK [*PearceJA@cardiff.ac.uk*](mailto:PearceJA@cardiff.ac.uk)*;*  ^5^Department of Earth Sciences, University of Durham, DH1 3LE, UK [*julie.prytulak@durham.ac.uk*](mailto:julie.prytulak@durham.ac.uk)*;* ^6^School of Geosciences, University of South Florida, 4202 East Fowler Ave., Tampa, FL 33604, USA [*ryan@mail.usf.edu*](mailto:ryan@mail.usf.edu)*;* ^7^Department of Geosciences, King Fahd University of Petroleum and Minerals, Dhahran 31261, Saudi Arabia [*sawhatta@gmail.com*](mailto:sawhatta@gmail.com)*;* ^8^Géosciences Montpellier, CNRS, Université de Montpellier, Montpellier, France [*Marguerite.Godard@umontpellier.fr*](mailto:Marguerite.Godard@umontpellier.fr)*;* ^9^School of Geosciences, University of Sydney, Sydney, 2006 NSW, Australia [*t.chapman@sydney.edu.au*](mailto:t.chapman@sydney.edu.au)*;* ^10^State Key Laboratory of Isotope Geochemistry, Guangzhou Institute of Geochemistry, Chinese Academy of Sciences, Guangzhou 510640, PR China [*hongyanli@gig.ac.cn*](mailto:hongyanli@gig.ac.cn)*;* ^11^Institute of Earth Sciences, NAWI Graz Geocenter, University of Graz, Austria [*walter.kurz@uni-graz.at*](mailto:walter.kurz@uni-graz.at)*;* ^12^Towson University, Dept. of Physics, Astronomy, and Geosciences, Towson University, Towson, MD 21252 USA [*wrnelson@towson.edu*](mailto:wrnelson@towson.edu)*;* ^13^CEOAS, Oregon State University, Corvallis, OR, USA [*dheaton@ceoas.oregonstate.edu*](mailto:dheaton@ceoas.oregonstate.edu)*;* ^14^Institut für Mineralogie, Universität zu Köln, Germany [*mkirchen@uni-koeln.de*](mailto:mkirchen@uni-koeln.de)*;* ^15^Kochi Institute for Core Sample Research, Japan Agency for Marine-Earth Science and Technology, Kochi, 783-8502 Japan [*shimmy@jamstec.go.jp*](mailto:shimmy@jamstec.go.jp)*;* ^16^Department of Science, Osaka University, Sumiyoshi-ku Osaka 558-8585 Japan [*sakuyama@sci.osaka-cu.ac.jp*](mailto:sakuyama@sci.osaka-cu.ac.jp)*;* ^17^Institute of Geology, Chinese Academy of Geological Science, Beijing 100037, P.R. China [*yibingli@cags.ac.cn*](mailto:yibingli@cags.ac.cn)*; ^18^Department of Geology, Centenary College, Shreveport, LA. 71104 USA* [*svetter@centenary.edu*](mailto:svetter@centenary.edu)*; §Current Address: Department of Geology, California State University, Sacramento, Sacramento, CA, USA*

1. **Table of Contents – Supplementary Data**
2. **2018GC007731-Supporting-Information (this file)**

This file contains supplemental figures (Figures S01 to S05), including major element characteristics, Nb-Th-Yb relations, rare earth element-based melting models, and extended multi-element plots (spider diagrams) that compare model-derived melt concentrations to observed concentrations.

1. **2018GC007731-DS01**

Dataset DS01: Whole rock chemical analyses of forearc basalts from IODP Expedition 352 by XRF and ICPMS. These data are also archived at PANGAEA Data Archiving & Publication (PDI-18813).

1. **2018GC007731 Data Table DS02**

Dataset DS02: P-T calculations for primitive lavas from IODP Expedition 352.

1. **Contents of this file**

Figures S01 to S05.

1. **Introduction**

This file contains supplemental figures, including major element characteristics, Nb-Th-Yb relations, rare earth element-based melting models, and extended multi-element plots (spider diagrams) that compare model-derived melt concentrations to observed concentrations. Concentration data from Data Supplemental *2018GC007731-DS01* (Excel Spreadsheet).


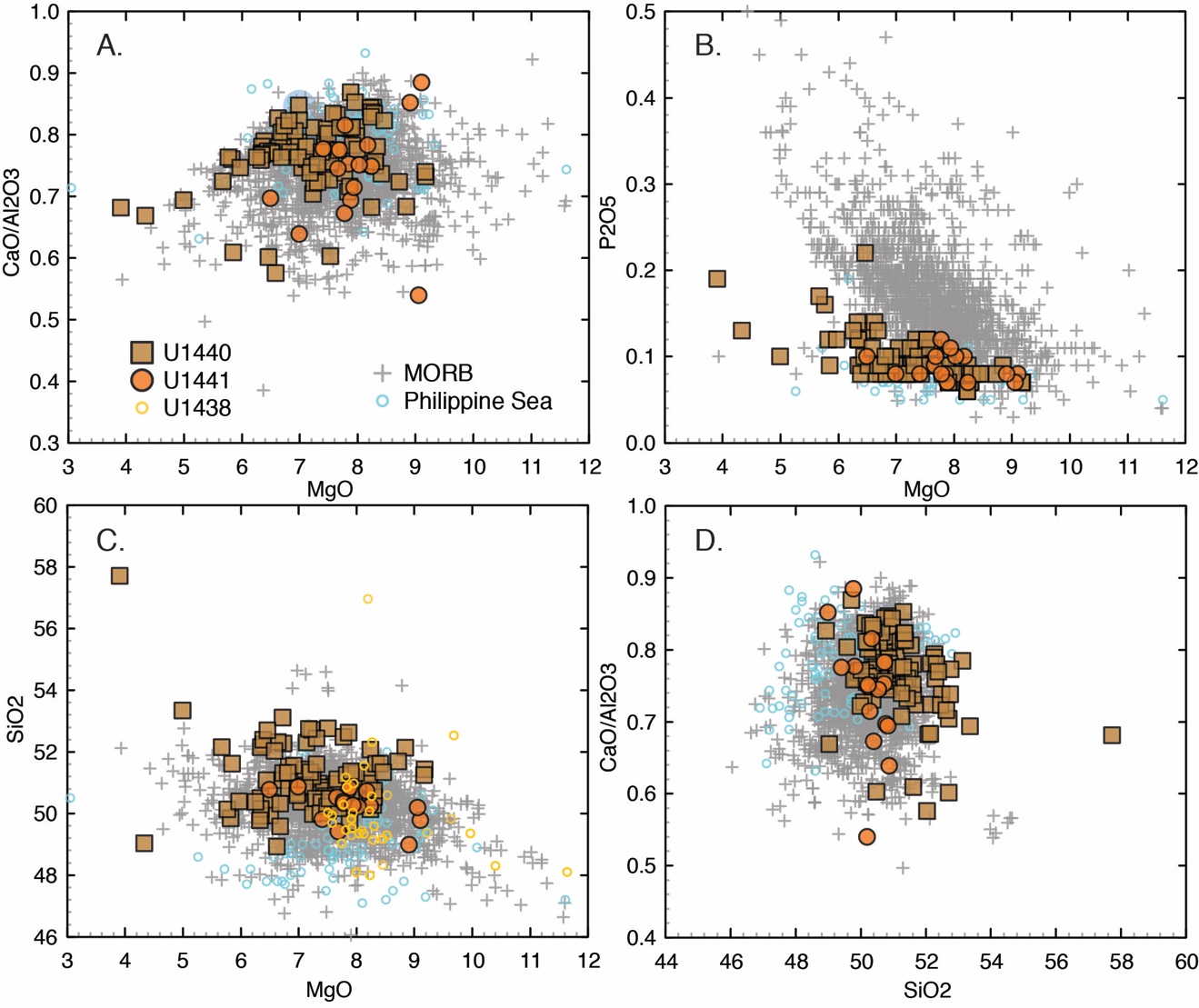


Figure S01. FAB are characterized by relatively high CaO/Al_2_O_3_ ratios, and low P_2_O_5_. They also have relatively high SiO_2_. SiO_2_ and CaO are within MORB array, but P_2_O_5_ is lower than most MORB. Global MORB data from PETDB, Philippine sea data from Mattey et al., 1980, and Site U1438 data from Hickey-Vargas et al, 2018.


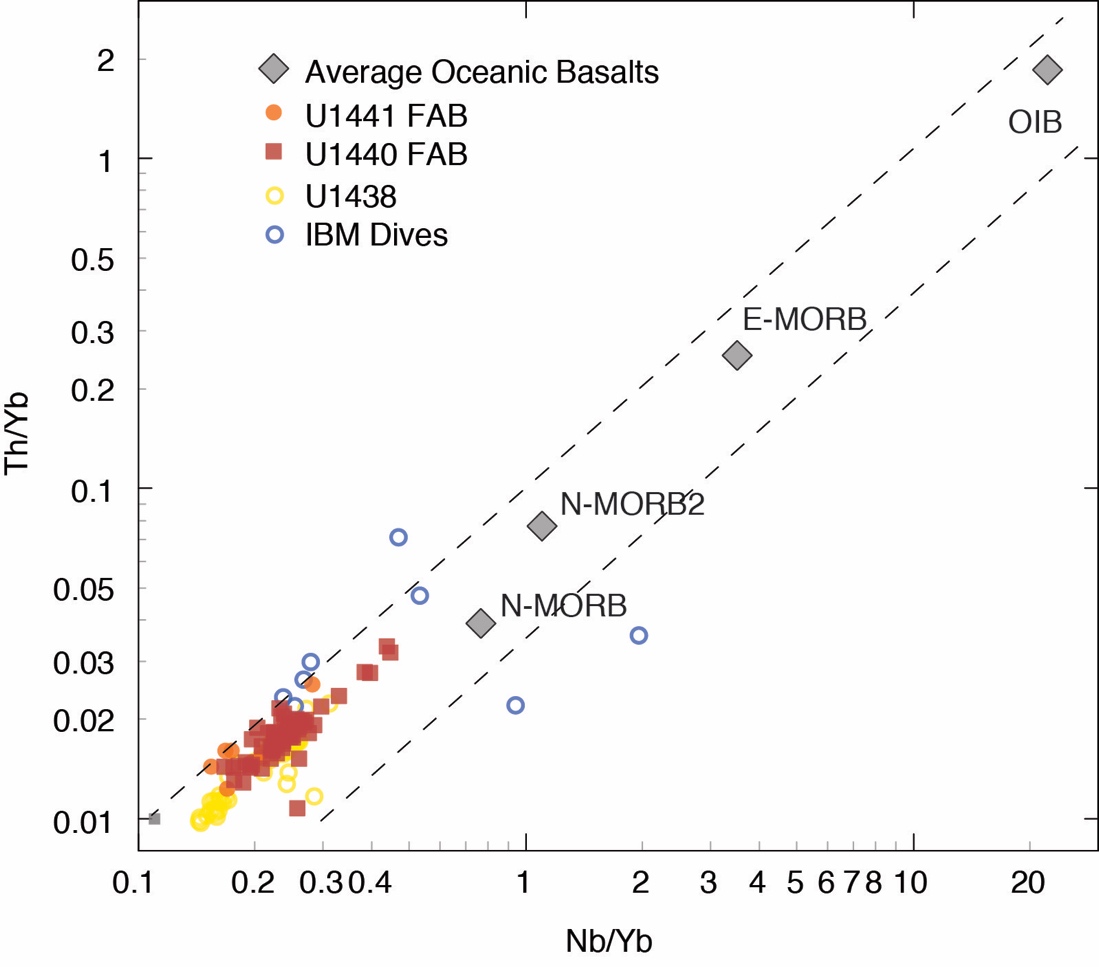


Figure S02. FAB are characterized by Nb/Yb and Th/Yb ratios that fall within the MORB array, but are significantly lower than most MORB. Our data are similar to depleted back-arc basin basalts of U1438 and to inner trench wall samples from DeBarri et al., 1999. MORB global array from Pearce, 1982, Philippine sea data from Mattey et al., 1980, and Site U1438 data from Hickey-Vargas et al, 2018.


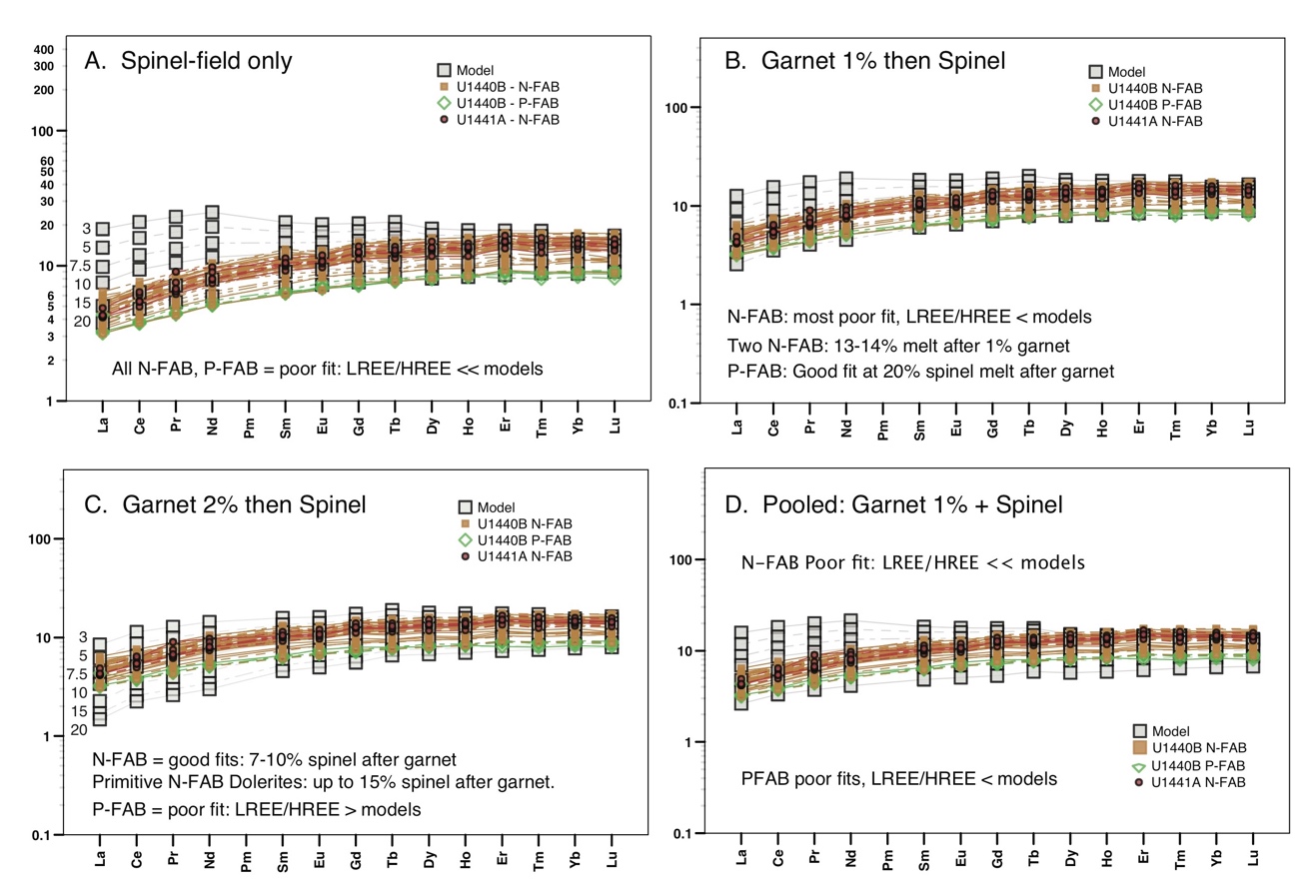


Figure S03. Rare earth element melting models for N-FAB (U1440, U1441) and P-FAB (U1440). All melt models (grey boxes): 3%, 5%, 7.5%, 10%, 15%, 20% and 23% spinel-field melt, with or without prior garnet-field melting. (A) Spinel-field only melting; (B) Extraction of 1% garnet-field melt before spinel-field melting; (C) Extraction of 2% garnet-field melt before spinel-field; (D) Pooled melts: 1% garnet-field melt pooled with spinel-field melts. Most N-FAB best fit with (C): 2% garnet-field melt extraction followed by 7-10% melting in the spinel field. Some N-FAB fit 1% garnet-field melt extraction followed by 10-15% melting in the spinel-field. P-FAB best fit with 1% garnet-field melt extraction followed by 20% melting in the spinel-field.

Melting model procedures including melt proportions and partition coefficients are described in the text.


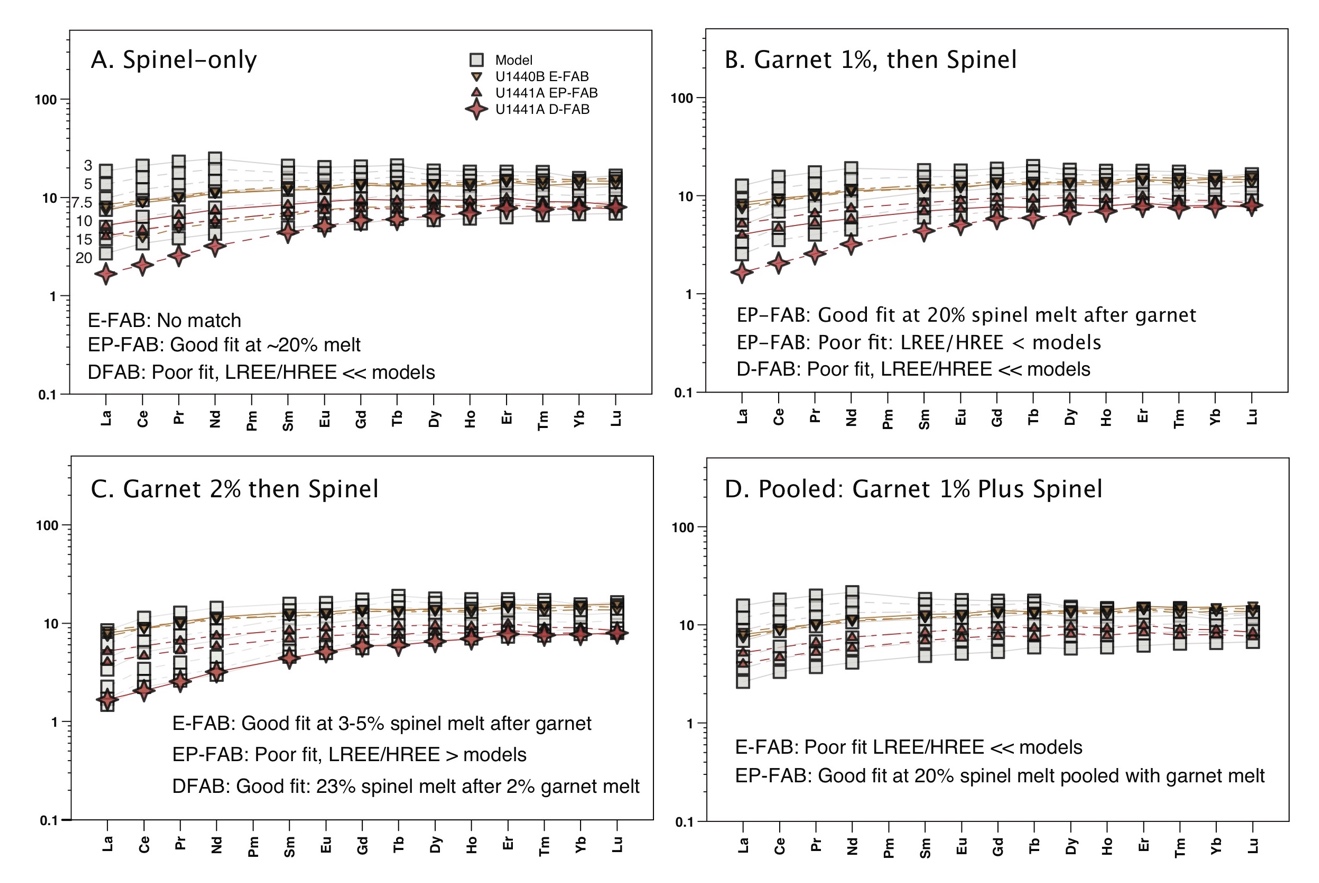


Figure S04. Rare earth element melting models for U1440 E-FAB, U1441 E-FAB, and D-FAB. All melt models (grey boxes): 3%, 5%, 7.5%, 10%, 15%, 20% and 23% spinel-field melt, with or without prior garnet-field melting. (A) Spinel-field only melting; (B) Extraction of 1% garnet-field melt before spinel-field melting; (C) Extraction of 2% garnet-field melt before spinel-field; (D) Pooled melts: 1% garnet-field melt pooled with spinel-field melts. U1440 E-FAB best fit with (C): 2% garnet-field melt extraction followed by 4-5% melting in the spinel field, or (B) 1% garnet-field melt extraction followed by 7-8% melting in the spinel field. U1441 E-FAB best fit with (A) 20% melting in the spinel field or (D) 1% garnet-field melt pooled with 20% or more spinel-field melts. D-FAB requires (C) 2% garnet-field melt extraction followed by 23% melting in the spinel field.

Melting model procedures including melt proportions and partition coefficients are described in the text.

**
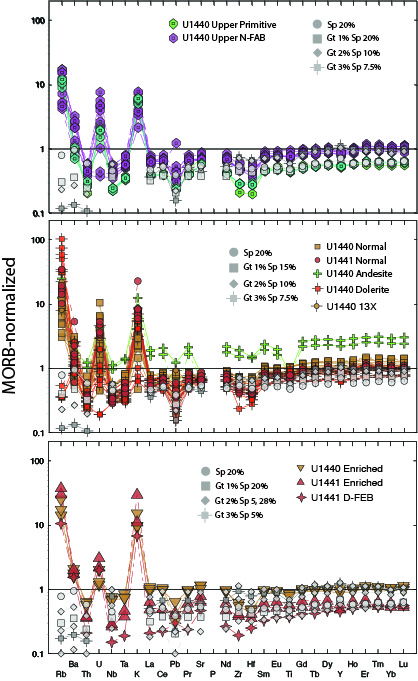
**

**Figure S05**. Extended multi-element melting models compared to observed data; melt models shown as gray symbols. (A) U1440 Upper N-FAB and P-FAB; (B) N-FAB (U1440, U1441) including andesite and dolerite; (C) U1440 E-FAB, U1441 EP-FAB, and D-FAB. Melt models shown in grey. In all cases, the fluid mobile elements (K, Rb, Ba, U) are enriched significantly above model values; this is mostly likely due to low-temperature alteration, although some enrichment may be reflect minor impact of slab fluids.
